# RUNX2 enhances vascular remodeling to promote endothelial proliferation in acute liver injury

**DOI:** 10.7150/ijbs.123366

**Published:** 2026-03-28

**Authors:** Jong-Min Lee, Yoon-Su Ha, Seung-Jun Lee, Hyun-Yi Kim, Anish Ashok Adpaikar, Eun-Jung Kim, Keishi Otsu, Xiangguo Che, Dai Hoon Han, Young Her, Je-Yong Choi, Seung-Jin Kim, Han-Sung Jung

**Affiliations:** 1 Division in Anatomy and Developmental Biology, Department of Oral Biology, Taste Research Center, Oral Science Research Center, BK21 FOUR Project, Yonsei University College of Dentistry, Seoul 03722, Republic of Korea.; 2 Department of Biochemistry, College of Natural Sciences, Global/Gangwon Innovative Biologics-Regional Leading Research Center (GIB-RLRC), BK21 FOUR Project, Kangwon National University, Chuncheon 24341, Republic of Korea.; 3 NGeneS Inc., Ansan-si 15495, Republic of Korea.; 4 Division of Developmental Biology and Regenerative Medicine, Department of Anatomy, Iwate Medical University, 1-1-1 Idaidori, Yahaba-cho, Shiwa-gun, Iwate 028-3694, Japan.; 5 Department of Biochemistry and Cell Biology, Cell and Matrix Research Institute, Skeletal Disease Analysis Center, School of Medicine, Kyungpook National University, Daegu 41944, Republic of Korea.; 6 Department of Surgery, Division of Hepato-biliary and Pancreatic Surgery, Yonsei University College of Medicine, Seoul 03722, Republic of Korea.; 7 Department of Dermatology, Kangwon National University Hospital, Kangwon National University School of Medicine, 156 Baengnyeong-ro, Chuncheon 24289, Republic of Korea.

**Keywords:** liver endothelial cells, endothelial progenitor cells, acute liver injury

## Abstract

**Background and Aims:**

The liver has a unique capacity for self-renewal, maintaining a proper liver-to-bodyweight ratio, which is essential for sustaining homeostasis. Regenerative process in the liver involves intricate communication between various cell types such as hepatocytes, hepatic stellate cells, endothelial cells, and inflammatory cells. Although the role of endothelial cells in liver regeneration has been extensively studied, detailed knowledge regarding specific endothelial cell-derived factors that promote the regeneration of liver endothelial cells (LECs) remains limited. This study aimed to identify the regenerative capacity of endothelial progenitor cells (EPCs) after acute liver injury.

**Methods:**

Thioacetamide (TAA) was used to induce acute liver injury. Bulk and single-cell RNA sequencing were analyzed to investigate changes in endothelial cells after TAA injection. *Runx2* heterozygous mice were analyzed to investigate the role of RUNX2 in endothelial regeneration.

**Results:**

TAA resulted in the delamination of LECs, which exhibited the highest regenerative capacity after three days of TAA injection. TAA increased the number of EPCs and RUNX2 was significantly enriched in the EPC population. Endothelial RUNX2 promotes regeneration by regulating its target genes such as *Lrp1*, *Gadd45b*, *Ptprj*, *Hmox1*, and *Junb*. In addition, *Runx2* haplodeficient mice exhibited diminished liver regenerative capacity compared with wild-type mice. RUNX2 is also expressed in the endothelial cells of patients with chronic liver diseases.

**Conclusion:**

These findings provide novel insights into the intricate mechanisms regulating LEC regeneration and highlight the pivotal role played by RUNX2 in vascular remodeling.

## 1. Introduction

The human liver accounts for approximately 2% of total body weight. Anatomically, it can be segmented into four lobes and functionally into eight segments, based on the pattern of blood supply and drainage through the portal and hepatic veins 1. Unlike other solid organs, the liver possesses the unique ability to regenerate, which helps to maintain the liver-to-bodyweight ratio essential for homeostasis 2. This regenerative process involves intricate communication between various cell types, such as hepatocytes, hepatic stellate cells (HSCs), endothelial cells, and inflammatory cells. Although a healthy liver remains quiescent primarily in terms of cell division, it can swiftly initiate cell cycle progression in response to injuries or surgical removal, thereby renewing its mass and functionality, by exhibiting highly synchronized responsiveness in both epithelial and non-parenchymal cells 1, 3.

Liver regeneration occurs via two distinct mechanisms that depend on the source of the regenerating hepatocytes 4, 5. First, following two-thirds partial hepatectomy (PHx), the residual hepatocytes undergo proliferation to restore the excised liver mass 6. Second, hepatocytes and biliary epithelial cells, known as cholangiocytes, possess the ability to de-differentiate into liver progenitor cells, which subsequently mature into hepatocytes 5. During PHx, quiescent HSCs transition into myofibroblasts and progenitor cells that are responsible for regenerating liver epithelial components 7. Resident Kupffer cells derived from monocytes play roles in tissue inflammation and damage 8. Furthermore, the interplay between the interleukin-1 family and Kupffer cells plays a pivotal role in influencing liver regeneration 9, 10.

Liver endothelial cells (LECs) are specialized endothelial cells that act as an interface between blood cells on one side and hepatocytes along with HSCs on the other side 11. In the physiological state, LECs remain quiescent and exhibit a low proliferation rate, and LECs play a pivotal role in liver regeneration after acute liver injury or PHx 12. This regenerative process involves the dynamic regulation of the balance between hepatocyte proliferation and vascular proliferation 13. Moreover, renewal of LECs involves three distinct cell types: mature LECs, resident endothelial progenitor cells (EPCs), and EPCs derived from the bone marrow 14, 15.

The role of circulating endothelial progenitor cells (EPCs) is well-established in promoting angiogenesis, particularly in treatment of cardiovascular conditions such as a coronary heart disease or ischemic injury 16, 17. Despite a considerable body of research about endothelial contribution to liver regeneration, our understanding of the factors that play a role in the association between endothelial cells and LEC regeneration needs to be elucidated.

Runt-related transcription factor 2 (RUNX2) is an essential for osteoblast differentiation and chondrocyte maturation 18. RUNX2 is known to be expressed in membranous and endochondral bone 19. RUNX2 has been extensively studied in the liver because it is a prognostic indicator of hepatocellular carcinoma (HCC) 20 and a critical factor in HSC activation during liver fibrosis 21. Although the Runt domain in RUNX2 has been recognized for its role in promoting neoangiogenesis in melanoma cells 22, little is known about the role of RUNX2 in liver endothelial regeneration 23.

In this study, thioacetamide (TAA), an organosulfur compound and a well-known hepatotoxin 24, was used to induce acute liver injury and to explore the processes operating within endothelial cells during LEC regeneration. Although TAA has been utilized to trigger acute liver injuries, such as oxidative stress and liver necrosis, as well as chronic injuries, such as fibrosis and cirrhosis 25-27, investigations of its impact on endothelial cells are limited. Our results indicated that TAA induced the delamination of endothelial cells, leading to severe liver damage, and that LECs showed remarkable regenerative capacity. RNA sequencing (RNA-seq) analysis indicated that the EPC population increased following TAA injection and that the expression of *Runx2* was highly enriched in this population. Endothelial RUNX2 promotes regeneration by regulating its target genes such as *Lrp1*, *Gadd45b*, *Ptprj*, *Hmox1*, and *Junb*. In addition, *Runx2* haplodeficient mice showed reduced regenerative capacity. In summary, these findings indicate that vascular remodeling involves regulation of angiogenesis and endothelial cell proliferation, in which RUNX2 plays a crucial role.

## 2. Methods

### 2.1 Animals and experimental model

All animal experiments were approved by the Yonsei University Health System Institutional Animal Care and Use Committee (YUHS-IACUC), in accordance with the Guide for the Care and Use of Laboratory Animals (National Research Council, USA). The animal study plan for these experiments (2021-0093) was reviewed and approved by our committee. All experiments were performed in accordance with the committee guidelines.

Adult C57BL/6 mice (purchased from Koatech Co. Pyeongtaek, Korea) were housed in a temperature-controlled room (22 °C) under artificial illumination (lights on from 05:00 to 17:00) and 55% relative humidity, with ad libitum access to food and water. Female mice were excluded to minimize variability associated with the estrous cycle. Mice from 9W were used in this study. *Runx2* haplodeficient mice were generated as previously described 28, 29. To establish the injury model, thioacetamide (TAA) was intraperitoneally injected into mice (50 mg/kg). All the operational procedures were performed under deep anesthesia.

Human liver biopsy specimens were obtained from patients with hepatocellular carcinoma (HCC) (n = 8), cirrhosis (n = 2), and nonalcoholic steatohepatitis (NASH) (n = 2) who underwent hepatectomy at the university-affiliated Severance Hospital, Yonsei University College of Medicine (Seoul, South Korea). Liver tissue was formalin-fixed for histological examination. The remaining tissue was snap-frozen in liquid nitrogen and stored at -80 °C for subsequent gene expression analysis. All participants provided written informed consent, and the study was approved by the independent institutional review board of Severance Hospital, Seoul, Korea (4-2016-0728).

### 2.2 Liver function test

For the liver function test, blood serum was obtained as follows. After collecting whole blood, the blood was allowed to clot by leaving it undisturbed at room temperature. The clot was removed by centrifugation at 2,000 xg for 10 min at 4 °C. The activities of serum alanine transaminase (ALT) and aspartate transaminase (AST) were measured using a GPT (ALT) assay kit (AM102, Asan Pharm, Co., Ltd, Korea) and GOT (AST) assay kit (AM103-K, Asan Pharm, Co., Ltd, Korea), according to the manufacturer's protocol.

### 2.3 Cell culture

Human Umbilical Vein Endothelial Cells (HUVECs; CC-2935, Lonza, Basel, Switzerland) were cultured in EGM™-2 BulletKit™ medium (CC-3162, Lonza) at 37 °C in a humidified atmosphere with 5% CO_2_. AML12 cells (American Type Culture Collection [ATCC]) were cultured in Dulbecco's Modified Eagle's/F12 Medium (DMEM/F12) at 37 °C in a humidified atmosphere with 5% CO_2._ RAW264.7 cells (ATCC) were cultured in DMEM at 37 °C in a humidified atmosphere with 5% CO_2._ HUVECs were transfected with *RUNX2* siRNA (SC-37145, Santa Cruz Biotechnology, Inc., USA), *CITED2* siRNA (SC-35959, Santa Cruz Biotechnology, Inc.), and *CXCR4* siRNA (SC-35421, Santa Cruz Biotechnology, Inc.) using FuGENE (E2311, Promega Corporation, WI, USA) according to the manufacturer's protocol. To manipulate the FGF and PI3K pathways, the cells were cultured in media containing 10 μM SU5402 (SML0443, Sigma-Aldrich, USA) and 50 μM LY294002 (#440202, Merck KGaA, Germany) for 24 h.

### 2.4 Immunohistochemistry and immunocytochemistry

Samples were fixed in 4% paraformaldehyde (PFA) in phosphate buffered saline (PBS) and embedded in paraffin using standard procedures. The specimen sections (5-μm thickness) were boiled in 10 mM citrate buffer (pH 6.0) and cooled at room temperature for 20 min. For von Willebrand Factor (vWF) and F4/80, sections were incubated in Proteinase K (10 μg/mL, AM2546, Thermo Fisher Scientific, USA) at 37℃ for 20 min. The cells were fixed in 4% PFA and permeabilized with 0.1% Triton X-100 in PBS. After blocking with normal goat serum, the sections were incubated with primary antibodies at 4 °C overnight. The primary antibodies used are listed in Table S1. The specimens were incubated with Alexa Fluor secondary antibodies (Invitrogen, OR, USA; 1:200) for 2 h at room temperature and counterstained with TO-PRO™-3 (T3605, Invitrogen; 1:1000). The sections and cells were examined under a confocal laser microscope (TCS SP8, Leica, Germany).

### 2.5 Western blot analysis

Mouse liver tissue extracts were fractionated by SDS-PAGE and transferred to polyvinylidene difluoride membranes (PVDF; Millipore) using a transfer apparatus, according to the manufacturer's protocols. After incubation with 5% skim milk in TBST for 1 h, the membrane was incubated with primary antibodies at 4 °C overnight. The primary antibodies used are listed in Table S1. Membranes were washed three times for 10 min and incubated with goat anti-rabbit IgG-HRP (7074S, Cell Signaling Technology, Inc., USA; 1:5000) or m-IgGĸ BP-HRP (SC-516102, Santa Cruz Biotechnology, Inc.; 1:5000) for 2 h. Blots were washed three times with TBST and developed using the ECL system (RPN2232, GE Healthcare Life Sciences, USA), according to the manufacturer's protocols.

### 2.6 Real time-quantitative polymerase chain reaction (RT-qPCR)

Total RNA was extracted using TRIzol^®^ reagent (#15596-026, Thermo Fisher Scientific). The extracts were reverse transcribed using Maxime RT PreMix (#25081, iNtRON, Korea). RT-qPCR was performed using a StepOnePlus Real-Time PCR System (Applied BioSystems, USA). The amplification program consisted of 40 cycles of denaturation at 95 °C for 15 s and annealing at 61 °C for 60 s. The expression levels of each gene were normalized against the housekeeping genes *B2m, GAPDH or 36B4*. Oligonucleotide primers used are listed in Table S2.

### 2.7 Liver endothelial cell isolation

Mouse livers were digested by retrograde perfusion with collagenase type 4 (LS004188, Worthington Biochemical Corporation, NJ, USA) through the portal vein. The dissociated cell mixture was placed into a 50 mL conical tube and centrifuged at 50 xg for 5 min to pellet the hepatocytes. The non-parenchymal cell-containing cell supernatant was further used to isolate LEC with FLK1 MicroBeads (130-097-346, Miltenyi Biotec, Germany), according to the manufacturer's protocols.

### 2.8 Statistical analysis

The graphic results are expressed as the mean ± standard deviation (SD). A GraphPad Prism 8 (GraphPad Software, San Diego, CA, USA) was used to analyze the data. Comparisons between two groups were performed using an unpaired two-tailed t-test. Comparisons of multiple groups were performed using one-way ANOVA followed by Tukey's multiple comparisons test. Statistical significance was set at *p* value < 0.05.

## 3. Results

### 3.1. TAA induces the endothelial delamination around the central vein of mouse liver

**Figure 1:**
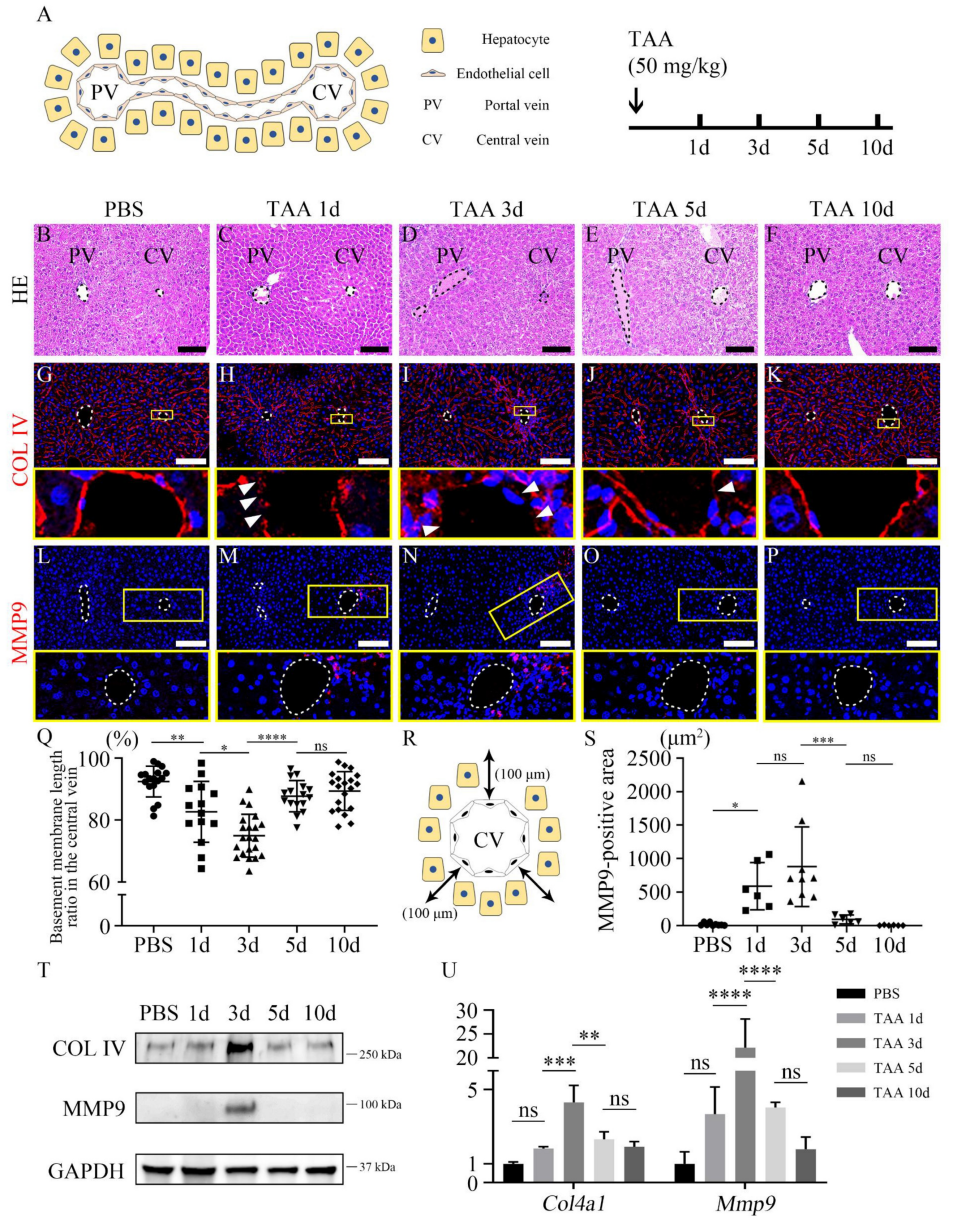
** Endothelial delamination following thioacetamide-induced liver injury.** (A) The liver is composed of its functional units, hepatic lobules, consisting of a portal triad, hepatocytes arranged between a capillary network, and a central vein. Thioacetamide is intraperitoneally injected into mice (50 mg/kg) for inducing liver injury. (B) After PBS injection (PBS group), a regular well-ordered arrangement of healthy hepatocytes and other non-parenchymal cells was observed. (C) After 1 day of TAA injection (TAA 1d), the injury is observed around the central vein. (D) In TAA 3d, inflammatory cells appear around the central vein. (E) In TAA 5d, the injured region recovers, while inflammatory cells are still observed around the central vein. (F) In TAA 10d, the liver tissue appears to have recovered to a level almost similar to that of PBS group. (G) In the PBS group, COL IV expression is uniformly observed in the entire endothelium of the liver. (H) In TAA 1d, COL IV is partially disconnected (arrowheads). (I) In TAA 3d, COL IV expression is mostly absent in the endothelium of the central vein, whereas it is significantly increased in the area surrounding the central vein (arrowheads). (J) In TAA 5d, the delamination of COL IV is partially restored, and its expression is reconnected (arrowhead). (K) In TAA 10d, COL IV expression is observed similarly to that in PBS group. (L) MMP9 is not expressed in PBS group. (M) In TAA 1d, MMP9-positive cells around the central vein are significantly increased. (N) In TAA 3d, the expression of MMP9 is highly observed around the central vein. (O) In TAA 5d, MMP9 is rarely found. (P) In TAA 10d, MMP9 expression is similar to that in PBS group. (Q) The basement membrane length ratio in the central vein is calculated as the ratio of the COL IV-positive total length minus the COL IV-negative delaminated region to the COL IV-positive total length in the central vein. The ratio is significantly decreased in TAA 1d and 3d livers compared to other groups. (R) Immunohistochemistry is used to quantify 100 μm from the central vein in a square area. (S) MMP9-positive area is significantly increased in TAA 1d and 3d liver compared to other groups. (T) Western blot analysis indicates that COL IV expression is dramatically increased in TAA 3d compared to other groups, and MMP9 expression is observed only in TAA 3d. (U) The mRNA expression level of *Col4a1* and *Mmp9* is significantly increased after 3 days of TAA. PV; portal vein, CV; central vein, TAA; thioacetamide. *p < 0.05, **p < 0.01, ***p < 0.001, ****p < 0.0001, ns; not significant. Scale bars; 100 μm.

TAA was intraperitoneally injected into the mice and livers were harvested at different time points after TAA injection to establish an injury model (Figure 1A). The overall liver function test indicated that the levels of alanine aminotransferase (ALT) and aspartate aminotransferase (AST) were significantly increased after 1 day of TAA injection (TAA 1d) and decreased from TAA 3d to TAA 10d. The level of ALT and AST in TAA 10d liver was comparable to that of PBS-injected group (Figures S1A and B). In the PBS group, hepatocytes and non-parenchymal cells, including endothelial cells, hepatic stellate cells, and Kupffer cells, exhibited well-organized arrangements within the hepatic lobules (Figure 1B, Figures S2A-D). However, following exposure to TAA 1d, a distinct area of injury became prominently evident surrounding the central vein (Figure 1C), albeit without visible inflammatory cells infiltration (Figure S2E). This injured region persisted, with inflammatory cells becoming concentrated around the central vein by TAA 3d (Figure 1D, Figure S2F). By TAA 5d, the extent of the injury had diminished, although inflammatory cells remained evident around the central vein (Figure 1E, Figure S2G). In TAA 10d, the liver appeared to have undergone recovery, displaying a similar structure to that of PBS-injected liver (Figure 1F, Figures S2H and I). To further investigate the phenomena occurring around the central vein, TEM was used to compare the central vein of the PBS-injected liver with that of the TAA-injected liver (Figures S3A-F). In PBS group, endothelial cells surrounding the lumen of the central vein and hepatocytes are separated by the basement membrane (Figure S3B). However, TAA led to endothelial delamination, as demonstrated by the discontinuity of basement membrane observed in TAA 1d (Figure S3C arrowheads). In TAA 3d, endothelial delamination was the most severe, and cell alignment was disrupted (Figure S3D arrowheads). In TAA 5d and 10d, the basement membrane and cell arrangement had recovered and reached a structural level similar to that of PBS group (Figures S3E and F). In addition, the expression of collagen IV (COL IV), an endothelial basement membrane marker, was examined using immunohistochemistry. In PBS group, COL IV expression was uniformly observed in endothelial cells (Figure 1G). However, in TAA 1d, COL IV was expressed in the discontinuous manner around the central vein (Figure 1H). COL IV expression was significantly decreased in TAA 3d and was discontinuous from the endothelial cells around the central vein (Figure 1I). In TAA 5d, COL IV expression was still disconnected but partially restored and reconnected (Figure 1J). Finally, the TAA 10d liver showed connected COL IV expression in the central vein (Figure 1K). Matrix metalloproteinase 9 (MMP9), also known as type IV collagenase, is a crucial factor in angiogenesis following endothelial injury. MMP9 was not observed in PBS-injected liver (Figure 1L). MMP9 expression dramatically increased at TAA 1d and 3d (Figures 1M and N). MMP9 expression was decreased in TAA 5d (Figure 1O) and absent in TAA 10d, similar to that in PBS group (Figure 1P). The ratio of basement membrane length in the central vein, determined by quantifying the length of the COL IV-expressing region to the total length of the central vein, progressively declined until TAA 3d and subsequently increased at TAA 5d (Figure 1Q). Additionally, MMP9 expression in a square area up to 100 μm away from the central vein was significantly increased in both TAA 1d and TAA 3d, compared to the other experimental groups (Figures 1R and S). In addition, western blot and RT-qPCR analyses of PBS- and TAA-injected liver indicated that the expression levels of COL IV and MMP9 in TAA 3d mice were significantly increased compared to those in the other groups (Figures 1T and U). Consistent with these findings, TAA injection also significantly increased hepatic mRNA expression of pro-inflammatory cytokines, including *Tnf-α, Il-1β, Il-6, Mcp-1 and Mip-1α,* as well as the anti-inflammatory cytokine* Il-10* (Figure S8A).

To further investigate the mechanism underlying TAA-induced MMP9 expression, we performed in vitro experiments. TAA treatment of AML12 hepatocytes increased the expression of pro-inflammatory cytokines and reduced the expression of antioxidant genes, indicating hepatocellular stress. Furthermore, conditioned medium from TAA-treated AML12 cells significantly increased Mmp9 expression in RAW264.7 macrophages compared with control conditioned medium (Figure S9A-C). These findings support the involvement of hepatocyte-derived inflammatory signals in MMP9 induction in macrophages following TAA exposure. Taken together, these findings suggest that TAA induced MMP9 expression around the central vein of the liver, resulting in subsequent endothelial delamination by degrading COL IV of the basement membrane.

### 3.2. Liver endothelial regenerative capacity is the highest at 3 days after TAA acute injury

**Figure 2:**
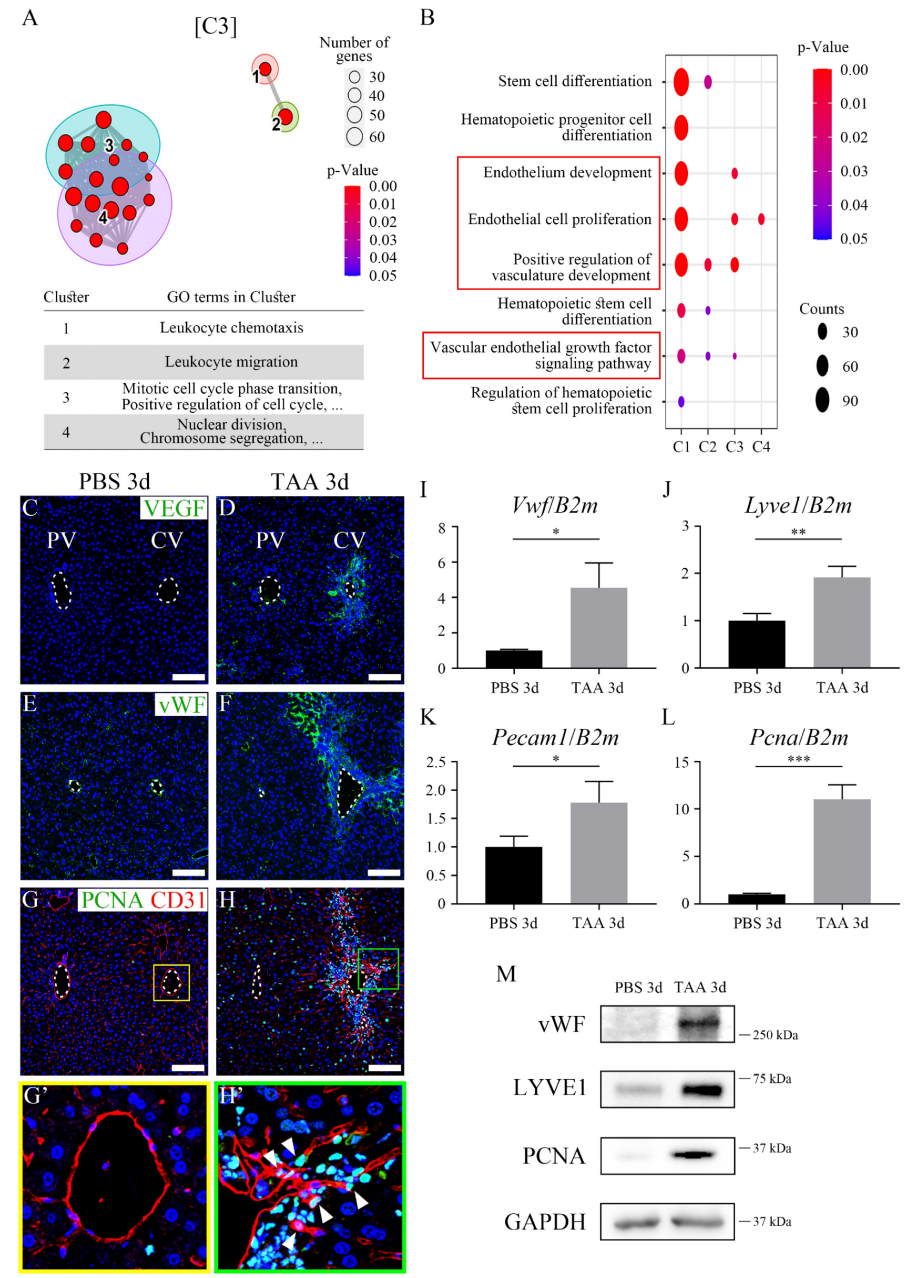
** Endothelial proliferation and angiogenesis after thioacetamide injection.** (A) Among the 4 clusters obtained via the time series analysis from bulk RNA-seq data, gene ontology (GO) terms associated with cell cycle and nuclear division are mostly observed in cluster 3 (C3). (B) GO terms associated with stem cell differentiation and endothelium development are significantly enriched in C2 and C3, respectively. (C) VEGF is not expressed in PBS 3d. (D) VEGF expression is observed in TAA 3d around the central vein. (E) Similar to VEGF expression, vWF expression is observed sparsely in the edge of the portal and central vein, and (F) dramatically increased in TAA 3d around the central vein. (G, G') PCNA is not co-expressed in CD31-positive endothelial cells of PBS 3d. (H, H') In TAA 3d, PCNA around the central vein is co-localized with CD31 (arrowheads). (I-L) The mRNA expression level of (I) *Vwf*, (J) *Lyve1*, (K) *Pecam1* and (L) *Pcna* is significantly increased in TAA 3d compared to those in PBS 3d. (M) In western blot analysis, the expression of vWF, LYVE1, and PCNA is increased in TAA 3d than that in PBS 3d. PV; portal vein, CV; central vein. *p < 0.05, **p < 0.01, ***p < 0.001. Scale bars; 100 μm.

To investigate the differences in endothelial cells following TAA-induced injury, endothelial cells of the central vein were sorted using FLK1^+^ magnetic beads while bulk RNA-seq was used to analyze PBS, TAA 1d, 3d, 5d, and 10d endothelial cells. Volcano plots indicated that gene expression in endothelial cells was significantly altered in TAA 3d (Figure S4A). In addition, time series analysis revealed that these genes could be grouped into four clusters according to their differential expression patterns (Figure S4B). Among them, the differential expression of cluster 1 (C1), which contained the most genes (8365), was considered insignificant. Clusters C2 and C3 showed increased expression in TAA 3d, whereas C4 showed decreasing expression. In addition, the extent of increase in C3 expression in TAA 3d was greater than that in C2. In C3, gene ontology (GO) terms associated with cell cycle, such as mitotic cell cycle phase transition, positive regulation of the cell cycle, nuclear division, and chromosome segregation, were mostly enriched (Figure 2A). Specifically, GO terms associated with endothelium development, endothelial cell proliferation, positive regulation of vasculature development, and vascular endothelial growth factor (VEGF) signaling pathway were highly enriched in C3 (Figure 2B).

The localization of cells involved in angiogenesis and proliferation in PBS 3d and TAA 3d liver tissues was examined by immunohistochemistry. Although VEGF, an angiogenesis marker, was not detected in the PBS 3d liver (Figure 2C), it was detected in proximity to the central vein in the TAA 3d liver (Figure 2D). Similarly, the expression of von Willebrand factor (vWF), another angiogenesis marker, was not observed in PBS 3d (Figure 2E), however, its expression dramatically increased in a broad region in the TAA 3d liver (Figure 2F). In addition, proliferating cell nuclear antigen (PCNA), a proliferation marker, was not detected in CD31-positive endothelial cells in PBS 3d liver (Figures 2G and G'), but was markedly increased around the central vein in TAA 3d liver and was partially co-localized with CD31-positive endothelial cells (Figures 2H and H' arrowheads). Although PCNA expression was also observed in other hepatic cell types after acute TAA injury, the increased co-localization of PCNA with CD31-positive cells at TAA 3d indicates enhanced endothelial proliferation. RT-qPCR analysis indicated that the mRNA expression levels of *Vwf*, *Platelet endothelial cell adhesion molecule* (*Pecam1*, endothelial marker), and *Pcna* were significantly increased in TAA 3d compared to those of PBS 3d (Figures 2I-L). A similar observation was made with western blot where protein expression levels of vWF, LYVE1 (LSEC marker), and PCNA were significantly increased in TAA 3d compared to those of PBS 3d (Figure 2M). Together, these results indicate that endothelial regeneration capacity was highly increased at 3 days of TAA injury.

### 3.3. Endothelial progenitor cell populations are increased after acute injury by TAA

**Figure 3:**
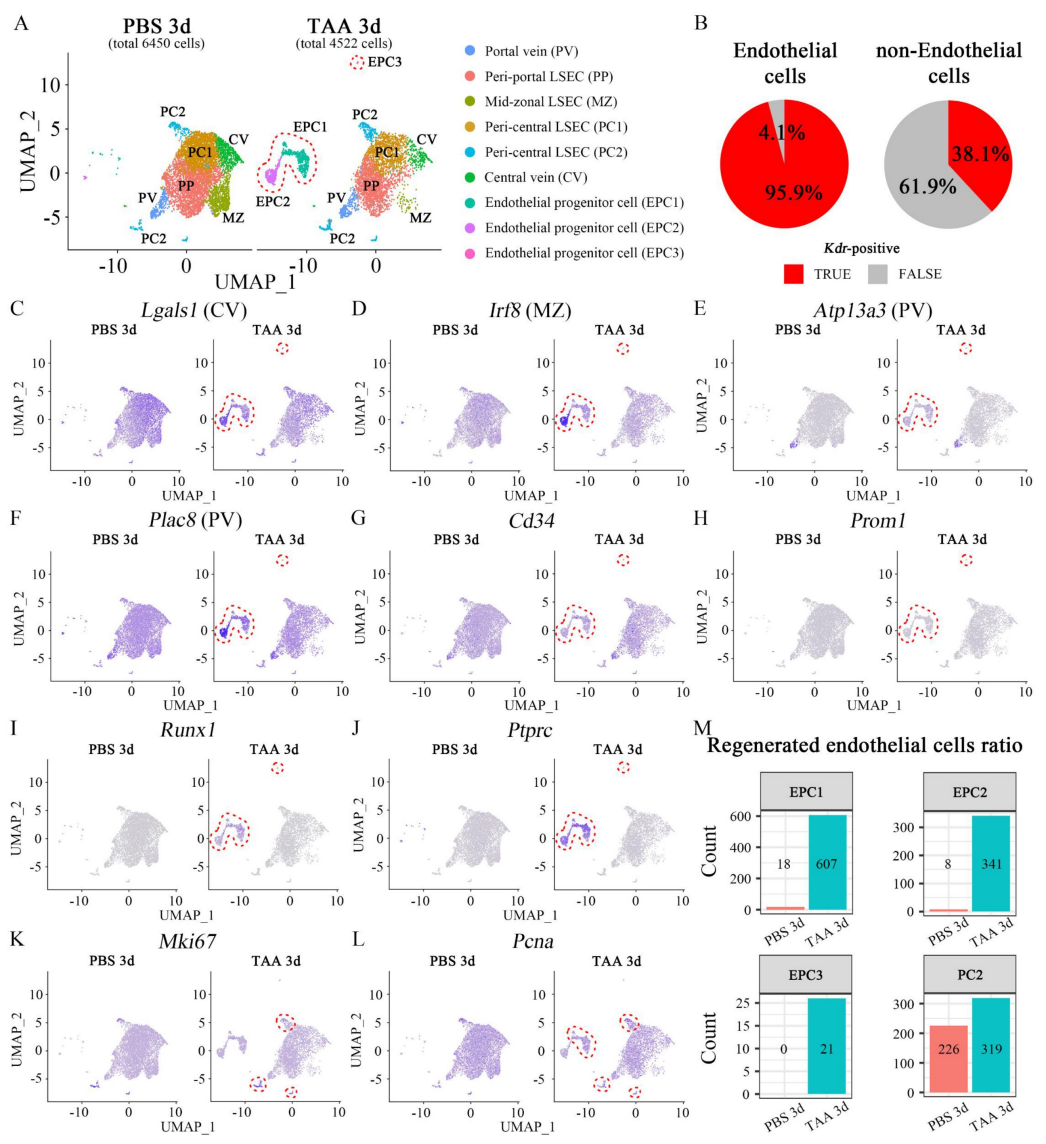
** Single-cell RNA-seq analysis of PBS- and TAA 3d liver endothelial cells.** (A) *Kdr*-expressing cells represent 6450 cells in PBS 3d and 4522 cells in TAA 3d, respectively. The subclustering of *Kdr*-expressing cells identifies 9 sub-clusters with portal vein (PV), peri-portal LSEC (PP), mid-zonal LSEC (MZ), peri-central LSEC (PC1, 2) and central vein (CV). Enriched endothelial cell clusters observed in TAA 3d, compared to PBS 3d, are identified as endothelial progenitor cells (EPC; EPC1, EPC2, EPC3; red dotted line). (B) 95.9% of endothelial cells and 38.1% of non-endothelial cells are *Kdr*-positive. (C-F) UMAP plots show that (C) *Lgals1* (PC2)-, (D) *Irf8* (MZ)-, (E) *Atp13a3* (PV)- and (F) *Plac8* (PV)-expressing cells are enriched in EPC clusters in TAA 3d. (G, H) The cells expressing typical endothelial progenitor markers, such as (G) *Cd34* and (H) *Prom1* are also enriched in EPC clusters in TAA 3d. (I) *Runx1*- and (J) *Ptprc*-expressing cells, which are involved in embryonic endothelial development and hematopoiesis, are enriched in EPC clusters in TAA 3d. (K) *Mki67*- and (L) *Pcna*-expressing cells are observed in clusters overall and enriched in the peri-central LSEC (PC2) cluster in TAA 3d. (M) Changes in cell numbers of EPC clusters and PC2 cluster are counted in PBS 3d and TAA 3d. In PBS 3d, 26 cells in EPC clusters (EPC1; 18 cells, EPC2; 8 cells, EPC3; 0 cells) and 226 cells in PC2 cluster are enriched. In TAA 3d, 969 cells in EPC clusters (EPC1; 607 cells, EPC2; 341 cells, EPC3; 21 cells) and 319 cells in PC2 clusters are enriched. In PBS 3d, the ratios of cells in EPC and PC2 cluster to the total cells are 0.40% and 3.50%, respectively. In TAA 3d, the ratios increase to 21.43% and 7.05%, respectively. The ratios of cells in the EPC and PC2 clusters to the total cells increase by 53.16 and 2.01 times, respectively.

Since the regenerative capacity was the highest at TAA 3d; PBS 3d and TAA 3d endothelial cells were analyzed via single-cell RNA-seq following FLK1^+^ MACS sorting. A UMAP plot identified seven clusters of macrophages, endothelial cells, hepatocytes, pericytes, B cells, gamma delta T cells, and fibroblasts (Figure S5A). *Kdr*, which encodes FLK1, was partially expressed in hepatocytes, macrophages, and pericytes as well as in overall endothelial cells (Figure S5B), and 63.4% of sorted cells were *Kdr*-positive (Figure S5C). Furthermore, *Kdr*-expressing cells were re-analyzed and nine sub-clusters of *Kdr*-expressing populations were identified. Based on zonal gene expression profiling of mouse LECs 30, these nine clusters were annotated as portal vein (PV), peri-portal LSEC (PP) 31, mid-zonal LSEC (MZ), two peri-central LSECs (PC1, 2), and central vein (CV) (Figure 3A). 95.9% of endothelial cells and 38.1% of non-endothelial cells were *Kdr*-positive (Figure 3B). UMAP plot indicated that *Kdr*-expressing cells were enriched in all clusters (Figure S5D) and *Ly6c1*-expressing monocytes were not highly enriched (Figure S5E). The marker genes representative of each cluster, which were significantly higher than that representative of the other clusters, are indicated by the dot plot (Figure S6A) and the UMAP plot (Figures S6B-G). The endothelial cells in three clusters in TAA 3d were found to be enriched compared to PBS 3d, and these were identified as EPC1, 2, and 3. *Kdr*-expressing cells represented 6450 cells in PBS 3d and 4522 cells in TAA 3d, respectively (Figure 3A). The genes significantly enriched in these three EPC clusters had all the characteristics of those enriched in endothelial cells in several zones (*Lgals1*; central vein, *Irf8*; mid-zonal, *Atp13a3*; portal vein, *Plac8*; portal vein) (Figures 3C-F). In addition, the expression of *Cd34*, a typical endothelial progenitor cell marker, was notably elevated in the EPC1, 2 and 3 cluster (Figure 3G), however, another marker, *Prom1* (*Cd133*), was not highly enriched (Figure 3H). *Runx1* has been reported to be involved in embryonic endothelial development, and *Ptprc*, which encodes CD45, is a marker of bone marrow-derived EPCs. The UMAP plot showed that *Runx1* and *Ptprc* were enriched in EPC1-3 clusters, indicating that these clusters played a role in EPCs function (Figures 3I and J). In addition, *Mki67* and *Pcna*, proliferation markers, were expressed in all clusters, albeit at low levels, but were enriched in the PC2 clusters in TAA 3d (Figures 3K and L), suggesting that the resident ECs in the peri-central region may also have regenerative capacity. Changes in the cell number within each cluster were examined to calculate the ratio of regenerated endothelial cells from EPCs or resident ECs. In PBS 3d, 26 cells were enriched in the EPC clusters (EPC1; 18 cells, EPC2; 8 cells, EPC3; 0 cells) and 226 cells in the PC2 cluster. In TAA 3d, 969 cells were enriched in EPC clusters (EPC1; 607 cells, EPC2; 341 cells, EPC3; 21 cells) and 319 cells in PC2 clusters. In PBS 3d, the ratios of cells in the EPC and PC2 clusters to the total cells were 0.40% and 3.50%, respectively. In TAA 3d, the ratios increased to 21.43% and 7.05%, respectively. The ratios of cells in the EPC and PC2 clusters to the total cells increased by 53.16 and 2.01 times, respectively (Figure 3M). This analysis indicated that the number of ECs regenerated from EPCs was dramatically higher than that from resident ECs following TAA-induced injury.

### 3.4. RUNX2 is expressed in endothelial progenitor cells and promotes liver endothelial regeneration

**Figure 4:**
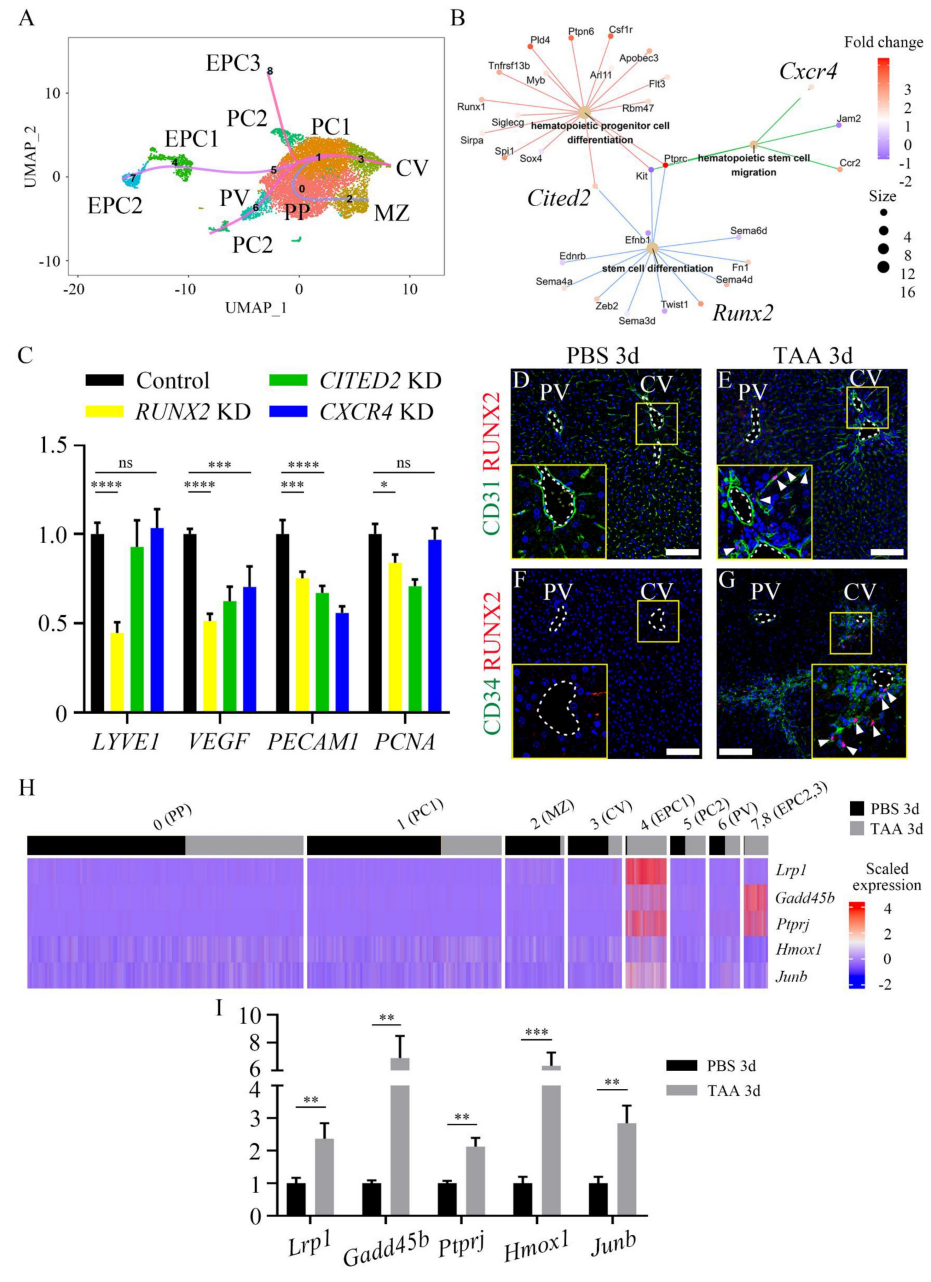
** RUNX2 is expressed in liver endothelial progenitor cells.** (A) The pseudo-time analysis reveals endothelial differentiation trajectories in both EPC and PC2 clusters toward the CV cluster. (B) Cnet plot indicates that several genes associated with GO terms, such as stem cell differentiation, hematopoietic stem cell migration, and hematopoietic progenitor cell differentiation, are increased in the EPC cluster. Among them, *Runx2*, *Cited2*, and *Cxcr4* are known to be expressed in endothelial cells. (C) The mRNA expression level of endothelial, angiogenesis and proliferation markers is significantly downregulated only in *RUNX2*-knockdown (KD) HUVECs, whereas that of *CITED2*- and *CXCR4*-KD HUVECs is not significantly downregulated. (D, F) RUNX2 expression is not detected in PBS 3d liver, (E, G) whereas it is observed to be increased around the central vein in TAA 3d liver, where it co-localized with (E) CD31-positive endothelial cells and (G) CD34-positive EPCs (arrowheads). (H) In cluster of EPC1, 2, and 3, the expression of RUNX2 target genes, such as *Lrp1*, *Gadd45b*, *Ptprj*, *Hmox1,* and *Junb,* is significantly increased. (I) In RT-qPCR analysis, the expression level of *Lrp1*, *Gadd45b*, *Ptprj*, *Hmox1*, and *Junb* in TAA 3d is significantly increased compared to that in PBS 3d liver. PV; portal vein, CV; central vein, PP; peri-portal LSEC, MZ; med-zonal LSEC, PC; peri-central LSEC, EPC; endothelial progenitor cell. *p < 0.05, **p < 0.01, ***p < 0.001, ****p < 0.0001, ns; not significant. Scale bars; 100 μm.

A pseudotime analysis was conducted to determine whether an EPC cluster can differentiate into endothelial cells in the injured central vein. This analysis revealed endothelial differentiation trajectories toward the CV cluster from either EPC clusters or PC2 cluster (Figure 4A), suggesting that EPC and resident pericentral endothelial populations have the capacity to differentiate into endothelial cells of the central vein. In addition, a cnet plot showed that the expression levels of several genes in GO terms, such as stem cell differentiation, hematopoietic stem cell migration, and hematopoietic progenitor cell differentiation, were increased in the EPC clusters. Among them, *Runx2, Cited2,* and *Cxcr4* were known to be expressed in endothelial cells (Figure 4B) 32-35. The mRNA expression levels of endothelial cell (*LYVE1, PECAM1*), angiogenesis (*VEGF*) and proliferation (*PCNA*) markers were significantly reduced in *RUNX2*-knockdown (KD) HUVECs; however, the expression level of *PECAM1* was even lower in *CITED2*-KD and *CXCR4*-KD HUVECs (Figure 4C). In addition, immunocytochemistry of Ki67 indicated that, of all the groups, only the proliferation of *RUNX2*-KD HUVECs was significantly lower than that of the control (Figures S7A-E). Expression of RUNX2 was not observed in the PBS 3d liver tissue (Figures 4D and 4F). Conversely, RUNX2 co-localization was found within CD31-positive endothelial cells around the central vein in TAA 3d (Figure 4E). In addition, RUNX2 also co-localized with CD34-positive EPCs (Figure 4G), suggesting that EPC express RUNX2 after TAA injury. Because RUNX2 is a transcription factor, the expression of its target genes was also examined. Of the previously selected putative target genes of RUNX2, those associated with the GO terms cell cycle, regulation of cell growth, DNA replication, G1/S transition of mitotic cell cycle, and blood vessel development were examined 36. The top five upregulated genes, *Lrp1*, *Gadd45b*, *Ptprj*, *Hmox1* and *Junb,* were enriched in the EPC clusters (Figure 4H). RT-qPCR analysis also indicated that the mRNA expression levels of *Lrp1*, *Gadd45b*, *Ptprj*, *Hmox1* and *Junb* in TAA 3d were significantly higher than those in PBS 3d (Figure 4I). Taken together, these results indicate that RUNX2 may play a role in EPC activation and promote angiogenesis and endothelial differentiation.

### 3.5. Haploinsufficiency of *Runx2* in mice delays liver endothelial regeneration

**Figure 5:**
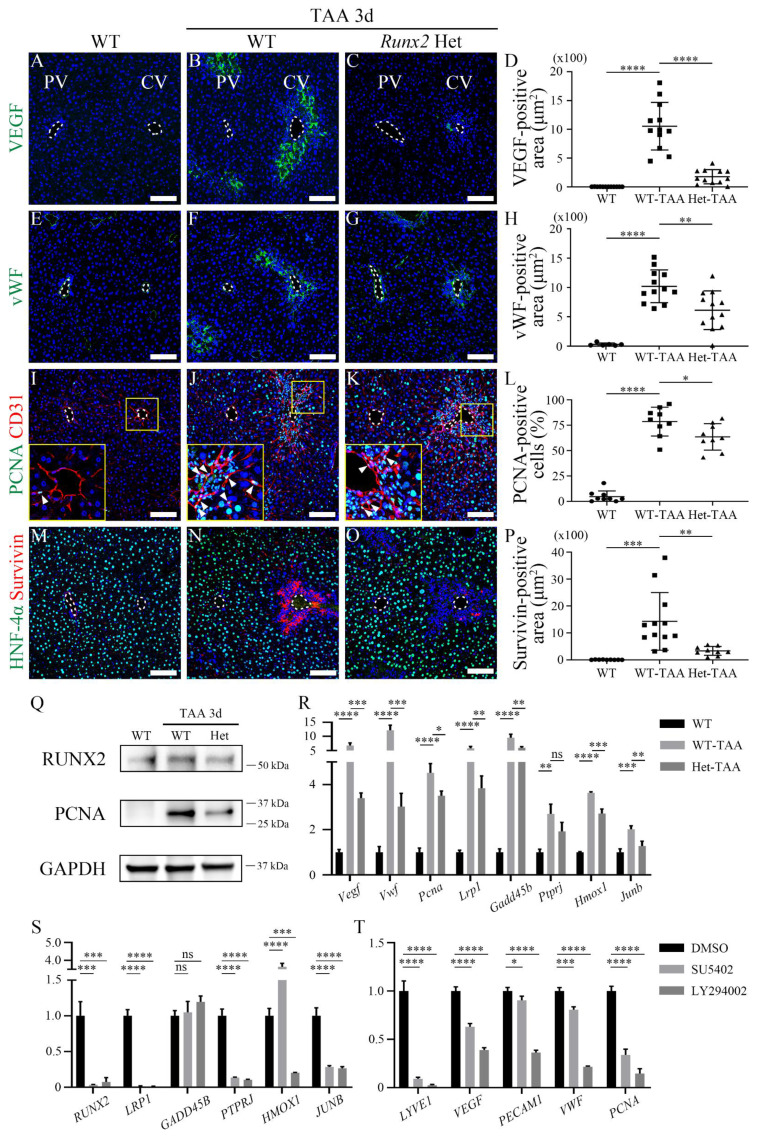
***Runx2* deletion delays the endothelial proliferation and angiogenesis in the liver.** Analysis of WT and *Runx2* heterozygous mice following TAA injection. The PBS injection for 3d group is indicated as WT. (A) VEGF is not expressed in the WT liver. (B) VEGF expression around the central vein of the WT-TAA is significantly increased. (C, D) However, VEGF expression in *Runx2* Het-TAA is lower than that in WT-TAA. (E) Similarly, vWF expression is not observed in the livers of WT mice. (F) Expression of vWF around the central vein of WT-TAA liver is increased, (G, H) but significantly reduced in *Runx2* Het-TAA compared to WT-TAA. (I) PCNA is rarely expressed in the WT liver (arrowhead). (J, K, L) In TAA 3d, PCNA and CD31 co-localized endothelial cells are increased around the central vein of WT-TAA (arrowheads) and *Runx2* Het-TAA (arrowheads). (M) In the WT liver, the expression of HNF-4α is observed overall, whereas Survivin is not expressed. (N) In WT-TAA, HNF-4α expression around the central vein is decreased, while Survivin expression in that region is increased. (O, P) Survivin-positive cells are found scarcely in *Runx2* Het-TAA. (Q) Western blot analysis indicates that the expression levels of RUNX2 and PCNA are increased in WT-TAA, but decreased in *Runx2* Het-TAA. (R) The mRNA expression levels of *Vegf*, *Vwf* and *Pcna* are significantly increased in WT-TAA livers compared to those in WT livers, but decreased in *Runx2* Het-TAA compared to WT-TAA. In addition, the mRNA expression levels of RUNX2 target genes such as *Lrp1*, *Gadd45b*, *Ptprj*, *Hmox1*, and *Junb* in WT-TAA are markedly increased compared to WT. RUNX2 target genes are downregulated in *Runx2* Het-TAA compared to WT-TAA, but *Ptprj* expression is not significant. (S, T) Following SU5402 (FGFR inhibitor) or LY294002 (PI3K inhibitor) treatment, (S) the mRNA expression of *RUNX2* and its target genes is decreased in HUVECs, except *GADD45B* and *HMOX1*. (T) Endothelial, angiogenic and proliferation markers are downregulated following SU5402 and LY294002 treatment. PV; portal vein, CV; central vein, TAA; thioacetamide. *p < 0.05, **p < 0.01, ***p < 0.001, ****p < 0.0001, ns; not significant. Scale bars; 100 μm.

As* Runx2* knockout or carboxy-terminus truncation mice exhibit embryonic lethality 31, 37, TAA was administered for 3d to induce liver injury in WT and *Runx2* heterozygous (Het) mice to identify the role of *Runx2* in endothelial regeneration. When the localization of markers related to angiogenesis and proliferation was examined, VEGF in TAA 3d WT (WT-TAA) mouse livers increased compared to that in PBS-injected WT (WT) livers (Figures 5A and B). In contrast, VEGF expression in *Runx2* Het-TAA liver was slightly decreased compared to that in WT-TAA liver (Figure 5C).

Quantification analysis also indicated that VEGF expression in *Runx2* Het-TAA livers was significantly lower than that in WT-TAA livers (Figure 5D). The vWF localization around the central vein of the WT-TAA liver was increased compared with that in the WT liver (Figures 5E and F), and was significantly decreased in the *Runx2* Het-TAA liver compared with that in the WT-TAA liver (Figures 5G and H). PCNA was rarely expressed in the central vein of the WT liver (Figure 5I). PCNA expression in the WT-TAA liver was dramatically increased and co-localized with endothelial cells (Figure 5J). PCNA expression in the *Runx2* Het-TAA liver was also increased compared to that in the WT liver, whereas the co-localization of CD31 and PCNA was decreased compared to that in the WT-TAA liver (Figure 5K). Quantification analysis indicated that the proportion of total PCNA-positive cells in the *Runx2* Het-TAA liver was significantly lower than that in the WT-TAA liver (Figure 5L). HNF-4α-positive cells around the central vein were decreased in both WT and *Runx2* Het mice, and the expression of the anti-apoptotic protein Survivin in the WT-TAA liver was significantly increased compared to that in *Runx2* Het-TAA livers (Figures 5M-P). Western blot and RT-qPCR analyses were used to validate the immunohistochemistry data. The protein expression of RUNX2 and PCNA in the *Runx2* Het-TAA liver was lower than that in the WT-TAA liver (Figure 5Q). The mRNA expression levels of *Vegf, Vwf* and *Pcna* were also significantly increased in WT-TAA livers but decreased in *Runx2* Het-TAA liver. In addition, the expression levels of RUNX2 target genes were examined. The upregulation of *Lrp1*, *Gadd45b*, *Ptprj*, *Hmox1*, and *Junb* in *Runx2* Het-TAA livers was consistently lower compared to WT-TAA livers, although the decrease observed in *Ptprj* was not significant (Figure 5R). Additionally, previously known pathway inhibitors were used to determine the signaling pathway that regulates endothelial regeneration via RUNX2 23, 31. SU5402, an FGFR inhibitor, and LY294002, a PI3K inhibitor, dramatically reduced *RUNX2* expression, following which the expression levels of RUNX2 target genes were reduced, except those of *GADD45B* and *HMOX1* (Figure 5S). To further examine whether RUNX2 directly regulates these target genes, ChIP-qPCR analysis was performed to assess RUNX2 binding to the promoter regions of candidate genes. However, no significant RUNX2 enrichment was detected at the promoter regions of *LRP1*, *GADD45B*, or *JUNB* under the conditions examined (Figure S10). Furthermore, the expression levels of the endothelial cell (*LYVE1*, *PECAM1*), angiogenesis (*VEGF*, *VWF*) and proliferation (*PCNA*) markers were decreased after SU5402 or LY294002 treatment compared to DMSO-treated control group (Figure 5T). Taken together, these results indicated that, in *Runx2* heterozygous mice, the expression levels of angiogenesis and proliferation markers, as well as those of *Runx2* target genes, were reduced, suggesting that loss of *Runx2* function may delay endothelial regeneration in the liver.

### 3.6. The endothelial RUNX2 in human chronic liver injuries

**Figure 6:**
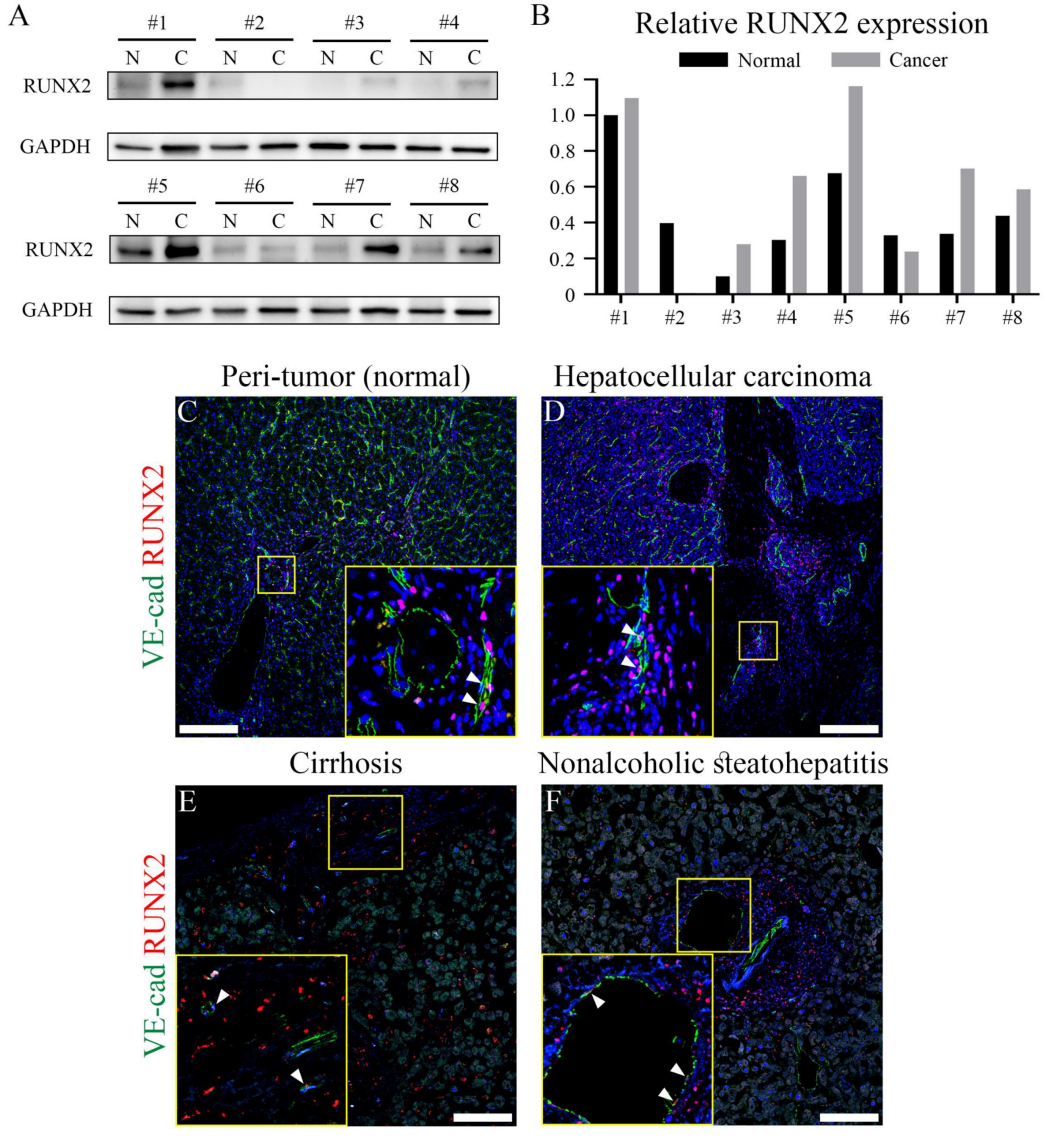
** Endothelial RUNX2 expression in human livers from patients with chronic liver disease.** (A) In western blotting analysis, the liver tissue from eight hepatocellular carcinoma patients RUNX2 expression is primarily increased in cancer, compared to that in peri-tumor (normal) liver. All hepatocellular carcinoma samples were derived from male patients, except sample #2. (B) Quantification via western blot indicates that relative RUNX2 expression is higher in cancer compared to that in peri-tumor of five hepatocellular carcinoma patients. (C-F) RUNX2 expression in hepatocellular carcinoma and peri-tumor is partially observed in endothelial cells (arrowheads). Endothelial cells in cirrhotic and NASH livers also express RUNX2 (arrowheads). N; peri-tumor (normal), C; cancer liver. Scale bars; C-D, 200 μm; E-F, 100 μm.

To determine whether endothelial RUNX2 contributes to the regeneration of various chronic liver injuries in humans, we examined RUNX2 expression in HCC, cirrhosis, and NASH tissues. Western blot of tumor tissue (C; cancer) and peri-tumor normal liver tissue (N; normal) obtained from eight HCC patients, showed that RUNX2 expression in cancer tissues was increased compared to that in normal tissues (Figure 6A), while quantification indicated that RUNX2 expression was significantly increased in six patients (Figure 6B). RUNX2 was primarily localized mostly around blood vessels and co-localized with several endothelial cells in the peri-tumor normal liver and HCC tissues (Figures 6C and D). Additionally, examination of RUNX2 expression in the liver of patients with cirrhosis and NASH indicated its expression in endothelial cells (Figures 6E and F). These results demonstrated that RUNX2 is strictly expressed in the LECs of humans with chronic liver injury for further understanding of regenerative capacity.

## 4. Discussion

Liver regeneration following acute injury is a beneficial process that has been studied extensively. Experimental injury models induced by partial hepatectomy or chemical injury have revealed extracellular and intracellular signaling pathways that return the liver to a size and weight equivalent to those prior to injury 2. As liver endothelial cells play a crucial role in maintaining metabolic and immune homeostasis, and actively contributing to disease pathophysiology 38, an investigation into the homeostasis and regeneration of liver endothelial cells is warranted.

TAA-induced acute injury affects all cell types of the liver, especially endothelial cells. TAA injection caused hepatic necrosis and accumulation of inflammatory cells in the necrotic area. Endothelial delamination was also observed in the present study. The disconnected expression of COL IV (the main component of the basement membrane) indicates the basement membrane of the endothelial cells was degraded and delaminated. The expression of MMP9 was increased in TAA 1d and 3d, indicating that the degradation of COL IV in the central vein was induced by MMP9 secreted from Kupffer cells 39. Western blot and RT-qPCR analyses showed that the expression of COL IV was increased; however, this increase was not derived from endothelial cells in the central vein, but rather from fibrotic tissue remodeling around the central vein, as previously reported during TAA-induced liver injury 40. Thus, TAA induces MMP9 expression to facilitate the delamination of endothelial cells in the central vein, resulting in injury.

The present study focused on the regeneration of LECs following acute injury. RNA-seq analysis, which identifies cellular changes, indicated that the significant change in gene expression of LECs was observed in TAA 3d. A subsequent time series analysis revealed that the altered genes were associated with endothelium development, endothelial cell proliferation, positive regulation of vasculature development, and the VEGF signaling pathway. Consistently, the protein and mRNA expression levels of genes involved in these pathways were markedly increased at TAA 3d. In parallel, PCNA expression was observed in multiple hepatic cell types, and increased PCNA/CD31 co-localization supported active LEC regeneration. Taken together, these findings suggest that the regenerative capacity of LECs is most prominent at three days following TAA injection. Although PCNA expression was also observed in multiple hepatic cell types at TAA 3d, reflecting overall liver regeneration following acute injury, the increased co-localization of PCNA with CD31-positive endothelial cells indicates that proliferative activity is also enhanced within liver endothelial cells during this regenerative phase.

ScRNA-seq is a powerful tool for identifying and classifying cell subsets, characterizing rare or small-cell subsets, and tracking cell differentiation along dynamic cell stages 41. In addition, the spatial distribution and zone-specific transcriptomic changes of LECs in humans and mice can be identified using scRNA-seq technology 30, 42, 43. In the process of LEC regeneration, the cells in the EPC cluster expressed *Cd34*, a typical EPC marker, and they also expressed all kinds of zone markers, including *Lgals1* for central vein, *IRF8* for mid-zonal LSEC, and *Atp13a3* and *Plac8* for portal vein. *Lgals1*, encoding Galectin-1, serves as a marker for cancer-associated fibroblasts and has been associated with poor prognosis in hepatocellular carcinoma (HCC) patients. Additionally, it functions as a nuclear matrix protein in osteoblasts 44, 45. *IRF8,* known for its expression in human hepatic B cells 43, has been identified as a novel therapeutic target in human hematopoietic diseases 46. Therefore, during the regeneration process, EPCs are differentiated into LECs. Still, since EPCs have all the characteristics of all zones, it is challenging to specify each EPC as a cell of a specific zone in this study.

*Runx2*, a gene that plays a key role during the early stages of development, has mainly been studied in bone. Previous studies have reported that RUNX2 promotes proliferation and cell cycle progression in human bone marrow endothelial cells 32. In addition, RUNX2 has been reported to regulate endothelial progenitor cell differentiation in response to mechanical stimulation 47. However, the role of RUNX2 in LEC regeneration following liver injury remains unclear. Our results indicate that RUNX2 expression was significantly increased in endothelial cells in TAA 3d. RUNX2 promotes liver endothelial regeneration following TAA-induced acute injury by activating endothelial proliferation and angiogenesis. Survivin-positive cells were found in WT-TAA, but were scarcely found in *Runx2* Het-TAA. Therefore, it is suggested that RUNX2 is considered to regulate Survivin expression, and liver regeneration. RUNX2 is regulated by various signaling pathways and molecules, including the FGF/PI3K signaling pathway and TWIST 23, 31, 48. Based on these studies, we investigated the involvement of these pathways in RUNX2 activation during LEC regeneration using pharmacological inhibitors. Inhibition of FGF and PI3K signaling markedly reduced RUNX2 expression and its downstream targets, implicating FGF and PI3K pathways in RUNX2 activation during LEC regeneration. To further investigate the mechanisms by which RUNX2 regulates downstream target gene expression during LEC regeneration, we performed ChIP-qPCR analysis to examine whether RUNX2 directly binds to the promoter regions of candidate target genes. However, no significant RUNX2 binding was detected at the examined promoter regions of *LRP1, GADD45B, and JUNB*, indicating that RUNX2 may not directly bind to these promoters under these conditions (Figure S10A and B). These results suggest that RUNX2 is likely to regulate these genes through indirect mechanisms. In line with this, a previous transcriptomic study identified *HMOX1* and *LRP1* as genes regulated by RUNX2 in human cell types in the absence of evidence for direct promoter binding 36. Previous studies have also reported indirect regulatory roles of RUNX2, suggesting that RUNX2 may regulate gene expression via modulation of chromatin accessibility, epigenetic mechanisms, or signaling pathways 49-53. Thus, RUNX2 may regulate downstream gene expression in a context-dependent or indirect manner during LEC regeneration. Further studies are needed to define RUNX2-dependent transcriptional regulation during LEC regeneration. As the early lethality in homozygous *Runx2* knockout mice, studies about adult *Runx2* knockout mice have not been suggested. Based on recent studies of bone defect model that used *Runx2* heterozygous mice 54, 55, we verified whether liver endothelial regeneration was affected. Runx2 heterozygous mice represent a partial reduction in Runx2 expression. In line with this, Runx2 protein levels were reduced in Runx2 Het-TAA livers, as confirmed by Western blot analysis (Figure 5Q). Our results indicated that TAA injection leads to reduction in the regenerative capacity of *Runx2* haplodeficient mice, suggesting that RUNX2 may be essential for liver endothelial regeneration. Further studies are needed to confirm the differentiation of ECs from EPCs using a lineage-tracing mouse model and to examine the mechanism of endothelial regeneration using an endothelial-specific Runx2-deleted mouse model.

Endothelial dysfunction is also associated with chronic liver injury. LECs undergo phenotypic changes and cellular senescence under disease conditions and display impaired autophagy in nonalcoholic fatty liver disease (NAFLD) 56. In NASH, LECs release inflammatory mediators and recruit inflammatory cells, thus promoting liver injury and inflammation 57. These altered LECs fail to maintain hepatic stellate cells quiescence and release fibrogenic mediators, thereby promoting liver fibrosis 58. Endothelial-specific TAZ deletion stimulates damage-induced liver fibrosis 59. The current study showed that RUNX2 is expressed in endothelial cells in various human chronic liver diseases, suggesting that endothelial RUNX2 may be a promising candidate to alleviate the progression of chronic liver diseases and resultant complications.

In conclusion, acute liver injury induced by TAA leads to MMP9-mediated endothelial delamination in the central vein. In response to endothelial delamination, LECs exhibited enhanced regenerative capacity within 3d, as evidenced by the increase of RUNX2/CD34-positive EPCs. RUNX2 in EPCs likely facilitates liver endothelial regeneration by modulating the transcription of target genes such as *Lrp1*, *Gadd45b*, *Ptprj*, *Hmox1*, and *Junb* in acute injury in the central vein*.* Taken together, this study could provide new insights into the mechanisms underlying the regulation of liver endothelial regeneration by RUNX2 and reveal novel pharmacological targets for treating liver diseases.

## Supplementary Material

Supplementary methods, figures and tables.
