# Supplementary material for: RUNX2 enhances vascular remodeling to promote endothelial proliferation in acute liver injury
Source: Int J Biol Sci. 2026 Mar 28;22(7):3909–24. doi: 10.7150/ijbs.123366 (PMC13086208; doi:10.7150/ijbs.123366)
Supplement: Supplementary file 1 — Supplementary methods, figures and tables. [file ijbsv22p3909s1.pdf]

Supplementary Methods

Transmission electron microscope (TEM)

Specimens were fixed for 12 h in 2% glutaraldehyde–2% paraformaldehyde in 0.1M phosphate buffer (pH 7.4) and washed in 0.1M phosphate buffer, post-fixed with 1% OsO_4 in 0.1M phosphate buffer for 2 h and dehydrated with an ascending ethanol series (50, 60, 70, 80, 90, 95, 100, 100%) for 10 min each, and infiltrated with propylene oxide for 10 min. Specimens were embedded with a Poly/Bed 812 kit (Polysciences), polymerized in an electron microscope oven (TD-700, DOSAKA, Japan) at 65℃ for 12 h. The block is equipped with a diamond knife in the Ultra-microtome (UC7, Leica Microsystems Ltd, Vienna, Austria), and is cut into 200 nm semi–thin section and stained toluidine blue for observation of optical microscope. The region of interest was then cut into 80 nm thin sections using the ultra-microtome, placed on copper grids, double stained with 5% uranyl acetate for 20 min and 3% Lead citrate for 7 min staining, and imaged with a transmission electron microscopy (HT7800, HITACHI, Tokyo, Japan) at the acceleration voltage of 80 kV equipped with a RC camera.

Bulk RNA-seq analysis

Libraries were prepared for 151bp paired-end sequencing using a TruSeq stranded mRNA Sample Preparation Kit (Illumina, CA, USA). Briefly, mRNA molecules were purified and fragmented from 1 μg of total RNA using oligo (dT) magnetic beads. The fragmented mRNAs were synthesized as single-stranded cDNAs via random hexamer priming. This was used as a template for second strand synthesis, to prepare double-

stranded cDNA. Following a sequential process of end repair, A-tailing and adapter ligation, cDNA libraries were amplified via PCR (Polymerase Chain Reaction). The quality of these cDNA libraries was evaluated using an Agilent 2100 BioAnalyzer (Agilent, CA, USA). They were quantified with a KAPA library quantification kit (Kapa Biosystems, MA, USA) according to the manufacturer's library quantification protocol. Following cluster amplification of denatured templates, paired end sequencing was conducted (2×151bp) using an Illumina NovaSeq6000 (Illumina, CA, USA).

Adapter sequences as well as ends of reads less than a Phred quality score of 20 were trimmed, and reads shorter than 50 bp were removed using cutadapt v.2.8 [1]. Filtered reads were mapped to the reference genome related to the species using the aligner STAR v.2.7.1a [2] following ENCODE standard options (refer to "Alignment" of "Help" section in the html report) with "-quantMode TranscriptomeSAM" option for estimation of transcriptome expression level.

Gene expression estimation was performed using RSEM v.1.3.1 [3], considering the direction of the reads which are corresponding to the library protocol using option --strandedness. To improve the accuracy of the measurement, "--estimate-rspd" option was applied. All other options were set to default values. To normalize sequencing depths among samples, FPKM and TPM values were calculated. Based on read counts estimated in the previous step, DEGs were identified using the R package, TCC v.1.26.0 [4]. The TCC package applies robust normalization strategies that enable tag count data to be compared. Normalization factors were calculated using the iterative DESeq2 [5]/edgeR [6] method. Q-value was calculated based on the p-value using the p.adjust function of the R package with default parameter settings. DEGs were identified based on a q-value

threshold less than 0.05 to correct errors caused by multiple-testing [7]. The GO database provides a set of hierarchical controlled vocabulary classified into 3 categories: Biological Process (BP), Cellular Component (CC) and Molecular Function (MF). The R package termed GOseq [8], involving the Wallenius non-central hypergeometric distribution, was used to conduct a GO based trend test, to functionally characterize the DEGs [9]. The genes that were selected ($p < 0.05$) following the test were regarded as statistically significant.

Single cell RNA-seq analysis

For single-cell RNA-seq analysis, liver endothelial cells were isolated from PBS-injected mice at day 3 (PBS 3d, n=1) and TAA-injected mice at day 3 (TAA 3d, n=1). A 10x Genomics Chromium platform was used to capture and barcode cells in order to generate single-cell Gel Beads-in-Emulsion (GEMs), following the manufacturer's protocol. Briefly, cell suspensions were loaded onto 10x Genomics Single Cell 30 Chips along with the reverse transcription master mix. During this step, cells were partitioned into GEMs along with gel beads coated with oligonucleotides. These oligonucleotides enable mRNA capture inside the droplets by 30 bp oligo-dT following cell lysis and provide barcodes to index cells (16 bp) as well as transcripts (12 bp UMI). Following reverse transcription, cDNAs with both barcodes were amplified, and a library was constructed using a Single Cell 3' Reagent Kit (v3.1 chemistry) for each sample. The resulting libraries were sequenced on an Illumina NovaSeq 6000 System in a 2 × 150 bp paired-end mode.

Sample demultiplexing, barcode processing, and UMI counting were performed using an official 10x Genomics pipeline Cell Ranger (v3.1.0).

(https://support.10xgenomics.com). Briefly, raw base call files generated by Illumina sequencers were demultiplexed into reads in FASTQ format using bcl2fastq developed by Illumina (https://github.com/brwnj/bcl2fastq). The raw reads were trimmed from the 3' end to obtain the recommended number of cycles for read pairs (Read1: 28 bp; Read2: 90 bp). The reads of each library were then processed separately using the "cellranger count" pipeline to generate a gene-barcode matrix for each library. During this step, the reads were aligned to a human reference genome (version: hg19). Cell barcodes and UMIs associated with the aligned reads were subjected to correction and filtering.

The scRNA-seq expression data were analyzed with Seurat v2.3.4 (PCA, Cluster, t-SNE and cluster). In brief, the Seurat object was generated from digital gene expression matrices. The parameter of "Filtercells" is mito-percentage (0 to 0.1). In the standard preprocessing workflow of Seurat, we selected 8706 variable genes following PCA. Then we performed cell cluster and t-SNE.

Preparation of AML12-derived conditioned medium

To induce hepatocellular stress, AML12 cells were treated with thioacetamide (TAA, 100 mM) for 24 h. After treatment, culture supernatants from PBS- or TAA-treated AML12 cells were collected and used as conditioned medium. RAW264.7 macrophages were then incubated with the conditioned medium for 24 h.

Chromatin Immunoprecipitation quantitative PCR (ChIP-qPCR) analysis

Chromatin immunoprecipitation (ChIP) was performed using a ChIP assay kit (17-295, MILLIPORE, USA) according to the manufacturer's instructions. Briefly, HUVECs were

crosslinked with formaldehyde, lysed, and chromatin was sonicated to an average fragment size of approximately 200-500 bp. One percent of the chromatin was used as input. For immunoprecipitation, chromatin was incubated with an anti-RUNX2 antibody or normal IgG as a negative control, followed by capture with protein A/G agarose beads (IP05, Calbiochem, USA). After washing, chromatin was eluted from the beads, crosslinks were reversed by incubation with NaCl at 65 ℃, and proteins were subsequently digested by proteinase K at 45 ℃. ChIP DNA was purified using a silica column-based PCR purification kit (Expin™ PCR SV DNA purification kit, GeneAll Biotechnology, Korea). Purified ChIP DNA and input DNA were analyzed by qPCR using EzAmp™ qPCR 2X Master Mix (Elpis Biotech, Korea) on a QuantStudio 1 Real-Time PCR system (Applied Biosystems, USA). The amplification program consisted of 40 cycles of denaturation at 95℃ for 15 s and annealing at 60℃ for 60 s. Input DNA was diluted 1:10 prior to qPCR analysis. Fold enrichment was calculated as the ratio of % input values obtained from RUNX2 immunoprecipitation relative to IgG controls. Oligonucleotide primers used are listed in Table S3. ChIP-qPCR experiments were performed in three independent biological replicates, and data are presented as mean ± standard error of the mean (SEM).

Supplementary Tables

Name	Catalog No.	Source	Dilution
**[IHC/ICC]**			
COL IV	ab6586	Abcam	1:200
MMP9	AB19016	Merck KGaA	1:200
VEGF	SC-365578	Santa Cruz Biotechnology, Inc.	1:200
vWF	ab6994	Abcam	1:200
PCNA	ab29	Abcam	1:300
CD31	28083-1-AP	Proteintech Group, Inc.	1:200
CD34	ab110643	Abcam	1:100
RUNX2	NBP1-77461	Novus Biologicals, LLC.	1:200
	SC-390351	Santa Cruz Biotechnology, Inc.	1:200
VE-cadherin	14-1449-82	Invitrogen	1:200
Ki67	ab16667	Abcam	1:200
HNF-4α	ab41898	Abcam	1:300
Survivin	2808S	Cell Signaling Technology, Inc.	1:300
GFAP	Z033429-2	Invitrogen	1:200
F4/80	ab6640	Abcam	1:100
**[WB/ChIP]**			
COL IV	ab6586	Abcam	1:1000
MMP9	AB19016	Merck KGaA	1:1500
vWF	ab6994	Abcam	1:2000
LYVE1	NB100-725	Novus Biologicals, LLC.	1:1000
PCNA	ab29	Abcam	1:500
RUNX2	NBP1-77461	Novus Biologicals, LLC.	1:1000
	ab192256	Abcam	1:1000
	SC-390351	Santa Cruz Biotechnology, Inc	3 μg/IP
GAPDH	SC-32233	Santa Cruz Biotechnology, Inc.	1:1000
normal IgG	SC-2025	Santa Cruz Biotechnology, Inc	3 μg/IP

Table S1. Antibodies for Immunohistochemistry (IHC), Western blot (WB), and Chromatin immunoprecipitation (ChIP) Table

Gene	Sequence (5'-3')
**[Mouse]**	
Col4a1	F-AAG GGA GAG CAA GGG GTC AG
	R-CCT GTT GGG GCA AAG TCT CC
Mmp9	F-GAC GGG TAT CCC TTC GAC GG
	R-GTG GTG GCG CAC CAG CGG TA
Vwf	F-CTC TTT GGG GAC GAC TTC ATC A
	R-GAC AGG CTC ATT CTC TTG CCA T
Lyve1	F-TGC CAC AAC TCA TCC GAC AC
	R-TGG AGT CAG GGG ATG AAG CC
Pecam1	F-GGA AGT GTC CTC CCT TGA GC
	R-GAG CCT TCC GTT CTT AGG GT
Pcna	F-TGC TGA CAT GGG ACA CTT AAA GTA TT
	R-CAA TGC CTA AGA TGC TTC CTC AT
Vegf	F-GTA ACG ATG AAG CCC TGG AGT G
	R-TTT TGA CCC TTT CCT CG
Lrp1	F-GGC GGT GTG ACA ACG ACA AT
	R-CGT GTG TCT CGT CGC TGT AG
Gadd45b	F-AGT CGT TCT GCT GCG ACA AT
	R-GTC TCG GGC TTC GGT TGT G
Ptprj	F-CCT GGA GCA ATG CAA ATG GC
	R-GGT ACC ATT GGC ATC CGG G
Hmox1	F-CCT TCC CGA ACA TCG ACA GC
	R-TGA GCA GGA AGG CGG TCT TA
Junb	F-AAG AGG AAC CGC AGA CCG TA
	R-CCG CTT TCG CTC CAC TTT GA

Tnf-α	F-AGG CTG CCC CGA CTA CGT
	R-GAC TTT CTC CTG GTA TGA GAT AGC AAA
Il-1β	F-TCG CTC AGG GTC ACA AGA AA
	R-CAT CAG AGG CAA GGA GGA AAA C
Il-6	F-ACA AGT CGG AGG CTT AAT TAC ACA T
	R-TTG CCA TTG CAC AAC TCT TTT C
Mip-1α	F-ACCTGGAACTGAATGCCTGAGA
	R-GCT TAT AGG AGA TGG AGC TAT GCA
Mcp1	F-TCT GGA CCC ATT CCT TCT TGG
	R-TCA GCC AGA TGC AGT TAA CGC
Il-10	F- TGG CCC AGA AAT CAA GGA GC
	R- CAG CAG ACT CAA TAC ACA CT
Ho-1	F- GAG CCT GAA TCG AGC AGA AC
	R- CCT TCA AGG CCT CAG ACA AA
Nrf2	F- TCT TGG AGT AAG TCG AGA AGT GT
	R- GTT GAA ACT GAG CGA AAA AGG C
Sod1	F- CCA GTG CAG GAC CTC ATT TT
	R- CAC CTT TGC CCA AGT CAT CT
36b4	F-AGA TTC GGG ATA TGC TGT TGG C
	R-TCG GGT CCT AGA CCA GTG TTC
B2m	F-CCT GGT CTT TCT GGT GCT TG
	R-CCG TTC AGC ATT TGG AT
**[Human]**	
LYVE1	F-TGG CTG GGT TGG AGA TGG AT
	R-TCA GGA CAC CCA CCC CAT TT

VEGF	F-AGA TCG AGT ACA TCT TCA AGC CAT
	R-CGT CAT TGC AGC AGC CC
PECAM1	F-GAG CAC CTC CAG CCA ACT TC
	R-GGG CTG GGA GAG CAT TTC AC
VWF	F-GCC AGA GCC TGC ACA TCA AT
	R-GCT GTT TCG GCA AAT GCA GG
PCNA	F-GTC CCA CGT CTC TTT GGT GC
	R-CGC CAA GGT ATC CGC GTT AT
RUNX2	F-GAG ATT TGT GGG CCG GAG TG
	R-ACT GAG GCG GTC AGA GAA CA
LRP1	F-CCG TAT GCT GGG TGC ATG TT
	R-CAC GAA GCC CTT TAG GCC AG
GADD45B	F-ACA TCA ACA TCG TGC GGG TG
	R-GTT GTT GCC CCG GCT TTC TT
PTPRJ	F-AGG AGG GAG CTG GCA ATT CT
	R-CAA ACT GTC CGA GAT GCC CC
HMOX1	F-CCA GTC TTC GCC CCT GTC TA
	R-GGC TGG TGT GTA GGG GAT GA
JUNB	F-GCG GCA GCT ACT TTT CTG GT
	R-GGG ACA ATC AGG CGT TCC AG
GAPDH	F-GAC GCT GGG GCT GGC ATT G
	R-GCT GGT GGT CCA GGG GTC

F, forward primer; R, reverse primer

Table S2. Primers for RT-qPCR

Gene	Sequence (5'-3')
**[Human]**	
LRP1 Site 1	F-ACA TGG TCC CTG CCC TCA AG
	R-TCT CGC TCT GCC TAT CTC TCC T
LRP1 Site 2	F-GAA GAG ACA CCG GGA AAG CA
	R-CGA AAC AGA CTT TAG ACA GCT TGA
GADD45B	F-TGG TCA GGT TGG TCT CGA AC
	R-GCA ATG TTT TAC GCA TGC AAC
JUNB	F-GAC TTC CAG ACC AGC CTA GG
	R-TGG GTA AAA GCG ATC CTC CTG

F, forward primer; R, reverse primer

Table S3. Primers for ChIP-qPCR

Supplementary Figures

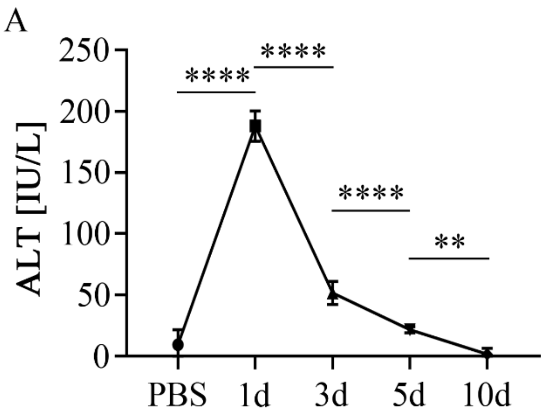

Figure S1. Alanine aminotransferase (ALT) and Aspartate aminotransferase (AST) levels in mouse liver after TAA injection

(A) Alanine aminotransferase (ALT) levels significantly increases in TAA 1d and decreases from TAA 3d to TAA 10d (n = 3). (B) Aspartate aminotransferase (AST) levels significantly increases in TAA 1d and decreases in TAA 3d (n = 3). The levels of (A) ALT and (B) AST in TAA 10d liver are comparable to those of the PBS-injected group. $^{**}p < 0.01$, $^{****}p < 0.0001$, ns; not significant.

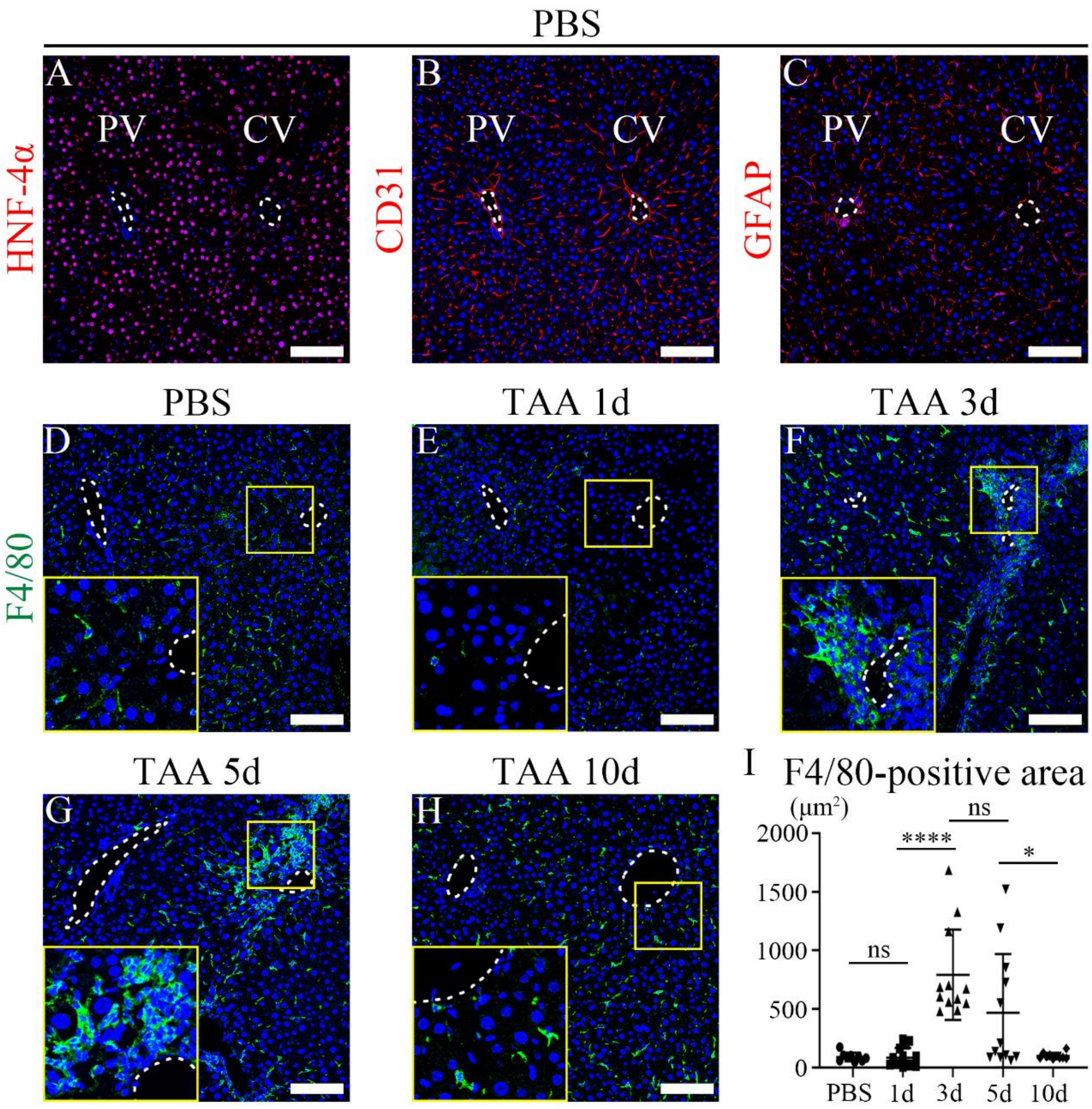

Figure S2. Expression of major liver cell type markers and the changes in inflammatory cells after TAA injection

(A-D) In the PBS-injected group, (A) hepatocytes, (B) endothelial cells, (C) hepatic stellate cells, and (D) Kupffer cells are examined by immunohistochemistry for HNF-4α, CD31, GFAP and F4/80, respectively. (E) F4/80 is sparsely expressed in TAA 1d. (F, G) F4/80-positive cells increased around the central vein of the liver in (F) TAA 3d and (G) TAA 5d. (H) F4/80-positive cells uniformly express in the liver of TAA 10d, similar to that of PBS

group. (I) F4/80-positive cells area around the central vein of the liver is significantly increased in TAA 3d and 5d compared to other groups. Scale bar; 100 μm.

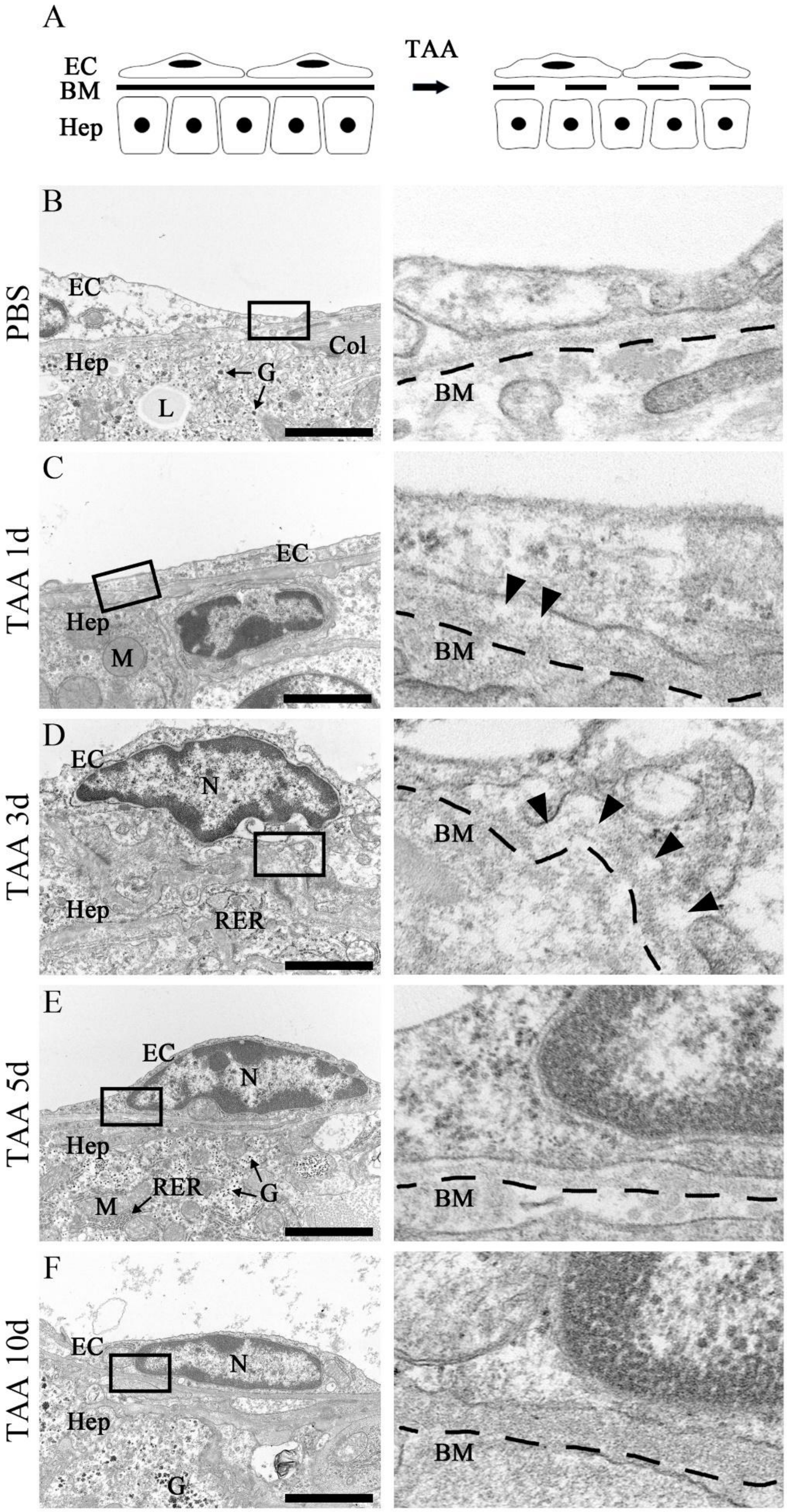
A
EC
BM
Hep
TAA
B
PBS
EC
Col
Hep
G
L
BM
C
TAA 1d
EC
Hep
M
BM
D
TAA 3d
EC
N
Hep
RER
BM
E
TAA 5d
EC
N
Hep
RER
M
G
BM
F
TAA 10d
EC
N
Hep
G
BM

Figure S3. Transmission electron microscope (TEM) analysis of mouse liver endothelial cells around the central vein after TAA injection

(A) Thioacetamide induces the delamination of the endothelial basement membrane. (B) In PBS-injected group, the endothelial cells and hepatocytes are well-aligned, and a basement membrane of the endothelial cells is observed between them (dotted line). (C) Delamination of the basement membrane is observed in TAA 1d (arrowheads). (D) The alignment of the basement membrane is irregular, and the degree of endothelial delamination in TAA 3d is higher than that in TAA 1d (arrowheads). (E) In TAA 5d, delamination of the basement membrane is no longer observed. (F) In TAA 10d, the arrangement of cells and the structure of the endothelial basement membrane are similar to those of PBS group. EC; endothelial cell, BM; basement membrane, Hep; hepatocyte, TAA; thioacetamide, Col; collagen fiber, G; glycogen, L; lipid droplet, M; mitochondria, N; nucleus, RER; rough endoplasmic reticulum. Scale bars; 2 μm.

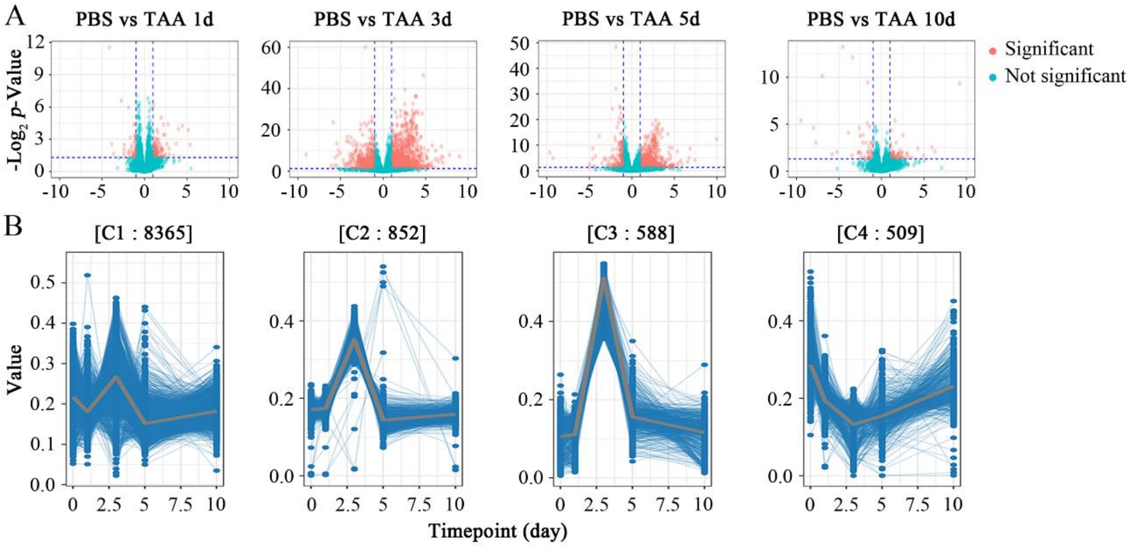

Figure S4. Bulk RNA-seq and time series analysis of endothelial cells between PBS- and TAA-injected mouse livers

(A) Bulk RNA-seq analysis of FLK1-positive endothelial cells from the PBS group, TAA 1, 3, 5 and 10d liver indicates that the most significant change in gene expression occurs in the endothelial cells of TAA 3d. (B) Time series analysis indicates 4 clusters, categorized based on patterns of gene expression changes. Cluster 3 (C3) has genes with the most upregulated expression changes in TAA 3d.

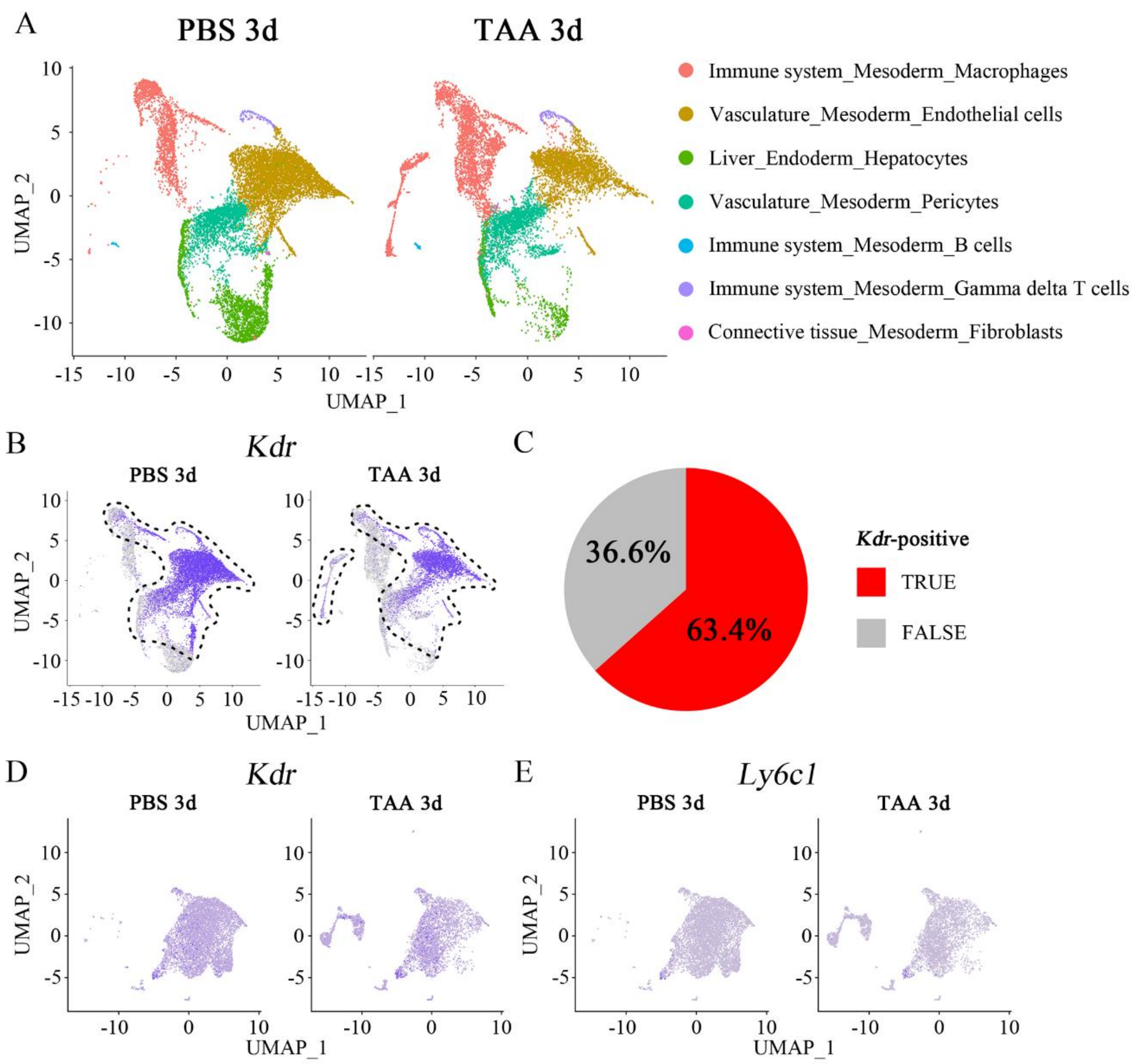

Figure S5. Single-cell RNA-seq analysis of liver endothelial cells between PBS- and TAA 3d groups

(A) Single cell RNA-seq analysis of PBS- and TAA 3d FLK1-positive liver endothelial cells identifies 7 clusters. (B) Expression of *Kdr* (FLK1) is observed in endothelial cells and other populations, including macrophages and pericytes. (C) Among FLK1-positive sorted cells, 63.4% are *Kdr*-positive. (D) After subclustering, *Kdr*-expressing cells are enriched in all clusters. (E) *Ly6c1*, a monocyte marker, is not expressed in endothelial progenitor cell clusters.

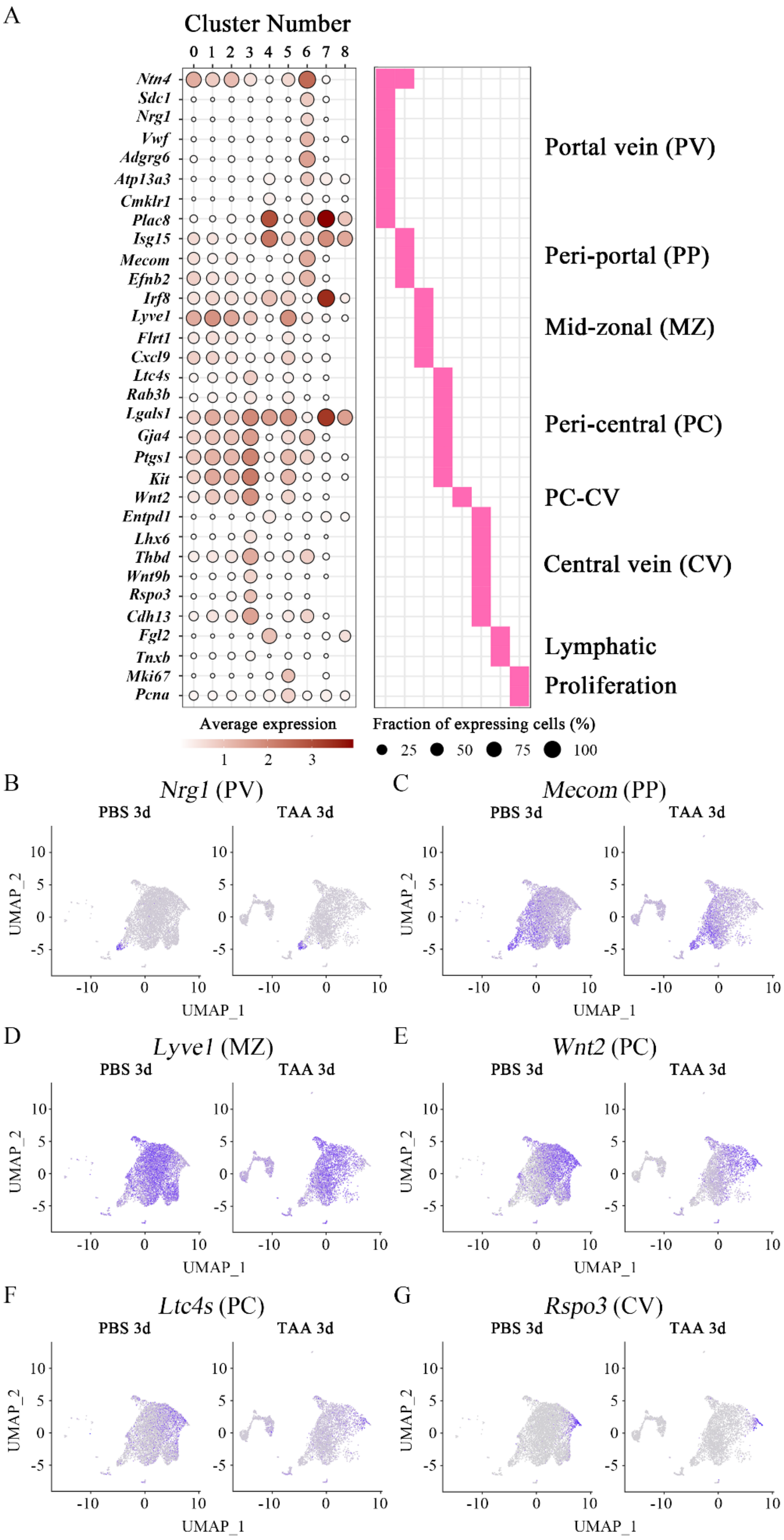

A
Cluster Number
0 1 2 3 4 5 6 7 8
Ntn4
Sdc1
Nrg1
Vwf
Adgrg6
Atp13a3
Cmklr1
Plac8
Isg15
Mecom
Efnb2
Irf8
Lyve1
Flrt1
Cxcl9
Ltc4s
Rab3b
Lgals1
Gja4
Ptgs1
Kit
Wnt2
Entpd1
Lhx6
Thbd
Wnt9b
Rspo3
Cdh13
Fgl2
Tnxb
Mki67
Pcna
Portal vein (PV)
Peri-portal (PP)
Mid-zonal (MZ)
Peri-central (PC)
PC-CV
Central vein (CV)
Lymphatic
Proliferation
Average expression
1 2 3
Fraction of expressing cells (%)
25 50 75 100
B
Nrg1 (PV)
C
Mecom (PP)
D
Lyve1 (MZ)
E
Wnt2 (PC)
F
Ltc4s (PC)
G
Rspo3 (CV)
PBS 3d
TAA 3d
UMAP_1
UMAP_2

Figure S6. Subclustering of liver endothelial cells and marker gene expression between PBS- and TAA 3d mouse liver endothelial cells

(A) Dot plot showing marker gene expression versus annotated clusters. The size of the dots represents the fraction of cells expressing the transcript and color intensity, the average expression level within a cluster. UMAP plots indicate that (B) *Nrg1* (portal vein marker)-positive cells are enriched in '6' cluster. (C) *Mecom* (peri-portal LSEC marker)-positive cells are enriched in '0' and '6' clusters. (D) *Lyve1* (mid-zonal LSEC marker)-positive cells are enriched in the '2' cluster. (E) *Wnt2* (peri-central LSEC and central vein marker)-positive cells are enriched in '1' and '3' clusters. (F) *Ltc4s* (peri-central LSEC marker)-positive cells are enriched in '5' and '3' clusters. (G) *Rspo3* (central vein marker)-positive cells are enriched in the '3' cluster.

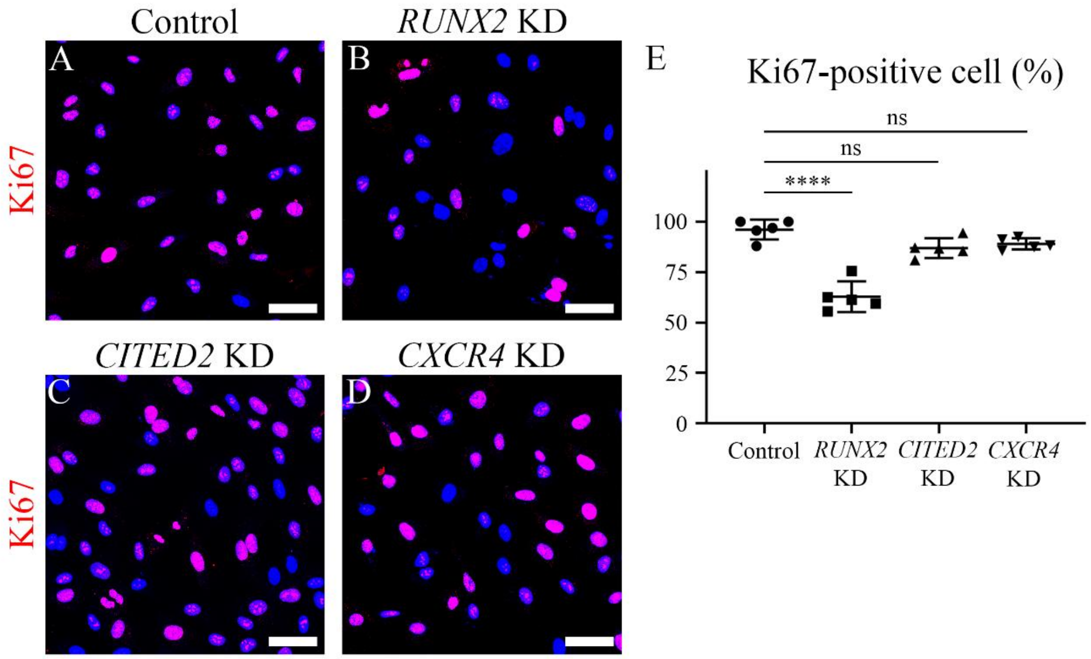

Figure S7. Quantification of Ki67-positive proliferative HUVECs after *RUNX2, CITED2,* and *CXCR4* knockdown

(A) Ki67 is expressed in most HUVECs in the control group. (B-D) Ki67-positive cells decrease following the knockdown of (B) *RUNX2*, (C) *CITED2*, and (D) *CXCR4* (KD) compared to control. (E) Quantification analysis indicates that Ki67-positive cells significantly decrease in *RUNX2* KD HUVECs, whereas no significant decrease is observed in *CITED2* KD and *CXCR4* KD cells (n = 5). ****$p < 0.0001$, ns; not significant. Scale bars; 50 μm.

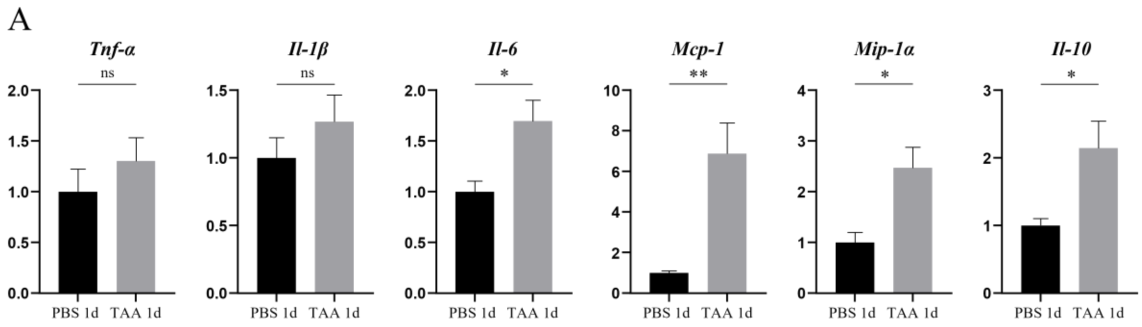

Figure S8. Acute inflammatory response in mouse liver at TAA 1d

(A) The mRNA expression levels of pro-inflammatory cytokines (*Tnf-α, Il-1β, Il-6, Mcp-1 and Mip-1α)* and the anti-inflammatory cytokine *(Il-10)* were measured by RT-qPCR at TAA 1d. *p < 0.05, **p < 0.01, ns; not significant.

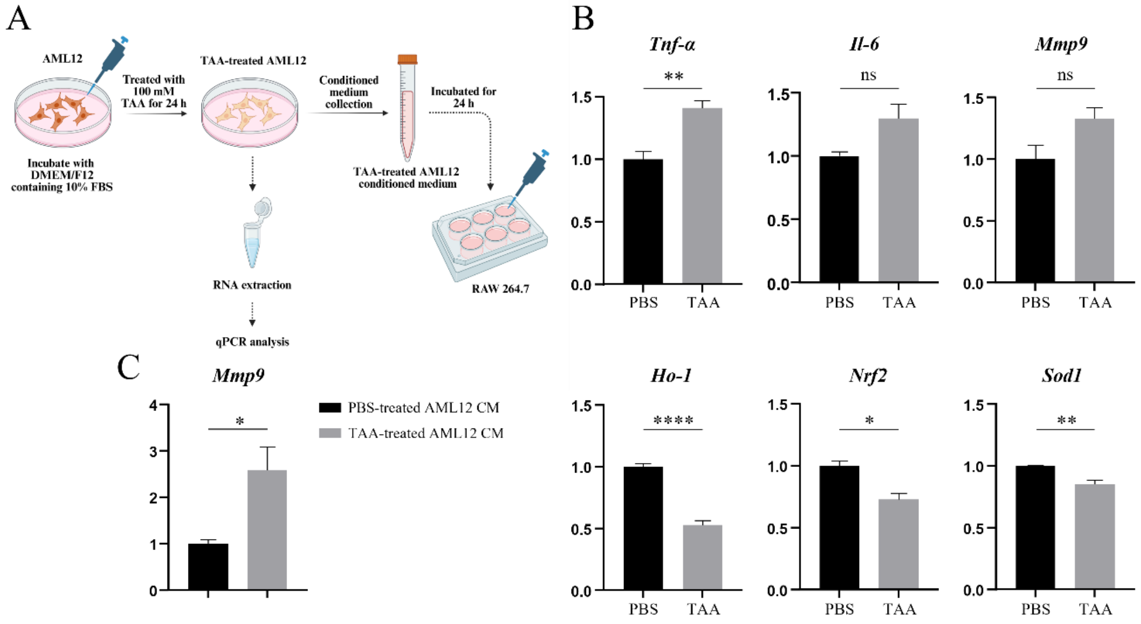

Figure S9. Hepatocyte-derived signals regulate MMP9 expression in macrophages

(A) Schematic illustration of the experimental design. (B) The mRNA expression levels of pro-inflammatory cytokines (*Tnf-α, Il-6*), *Mmp9*, and antioxidant-related genes (*Ho-1, Nrf2, and Sod1*) in AML12 cells were measured by RT-qPCR. (C) RAW264.7 macrophages were incubated with conditioned medium from PBS- or TAA-treated AML12 cells, and MMP9 expression was measured by RT-qPCR. *$p < 0.05$, **$p < 0.01$, ****$p < 0.0001$, ns; not significant.

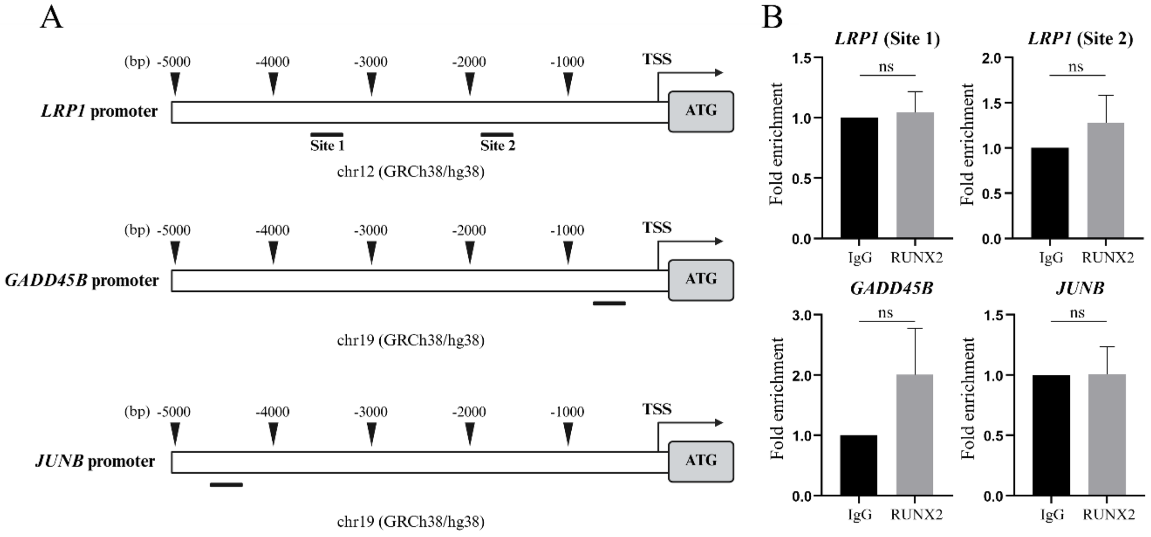

Figure S10. ChIP-qPCR analysis of RUNX2 enrichment at candidate target gene promoters

(A) Schematic representation of the promoter regions (-5 kb to the transcription start site [TSS]) of *LRP1, GADD45B, and JUNB*. Black bars indicate the regions analyzed by ChIP-qPCR. Sites 1 and 2 represent two distinct regions within the *LRP1* promoter. (B) ChIP-qPCR analysis of RUNX2 enrichment at the indicated promoter regions of *LRP1, GADD45B*, and *JUNB*. Data are presented as fold enrichment normalized to input DNA. Error bars represent the standard error of the mean (SEM) from three independent biological replicates. ns; not significant.

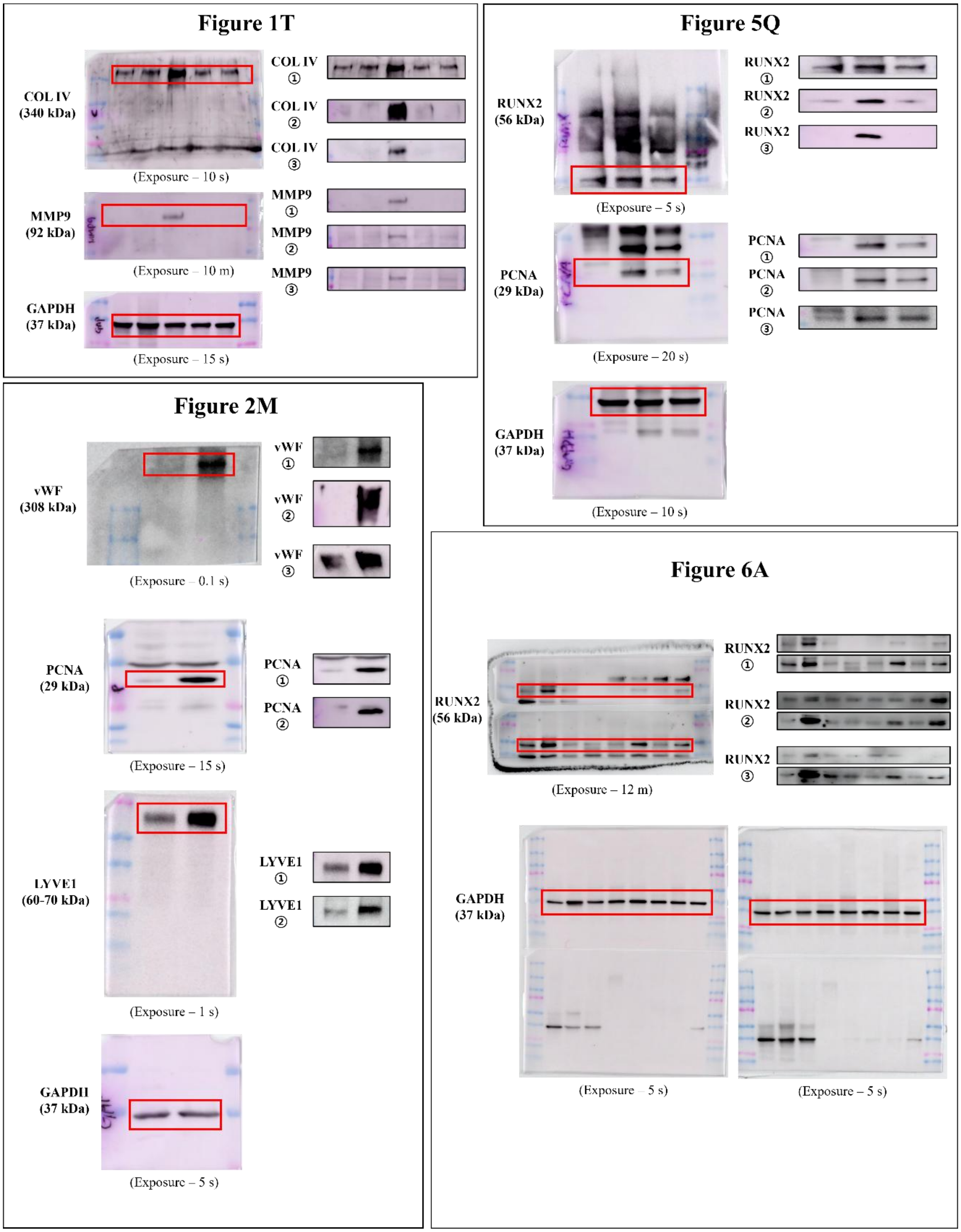

Figure S11. Original, unprocessed Western blot images

References

1. Martin M. Cutadapt removes adapter sequences from high-throughput sequencing reads. EMBnet J. 2011; 17: 10-2.
2. Dobin A, Davis CA, Schlesinger F, Drenkow J, Zaleski C, Jha S, et al. STAR: ultrafast universal RNA-seq aligner. Bioinformatics. 2013; 29: 15-21.
3. Li B, Dewey CN. RSEM: accurate transcript quantification from RNA-Seq data with or without a reference genome. BMC Bioinformatics. 2011; 12: 323.
4. Sun J, Nishiyama T, Shimizu K, Kadota K. TCC: an R package for comparing tag count data with robust normalization strategies. BMC Bioinformatics. 2013; 14: 219.
5. Love MI, Huber W, Anders S. Moderated estimation of fold change and dispersion for RNA-seq data with DESeq2. Genome Biol. 2014; 15: 550.
6. Robinson MD, McCarthy DJ, Smyth GK. edgeR: a Bioconductor package for differential expression analysis of digital gene expression data. Bioinformatics. 2010; 26: 139-40.
7. Benjamini Y, Hochberg Y. Controlling the false discovery rate: a practical and powerful approach to multiple testing. J R Stat Soc B. 1995; 57: 289-300.
8. Young MD, Wakefield MJ, Smyth GK, Oshlack A. Gene ontology analysis for RNA-seq: accounting for selection bias. Genome Biol. 2010; 11: R14.
9. Wallenius KT. Biased sampling; the noncentral hypergeometric probability distribution. Stanford, USA: Stanford University Applied Mathematics and Statistics Labs; 1963.
